# CRISPR-Cas9 mediated mutation in *GRAIN WIDTH and WEIGHT2* (*GW2*) locus improves aleurone layer and grain nutritional quality in rice

**DOI:** 10.1038/s41598-021-00828-z

**Published:** 2021-11-09

**Authors:** V. Mohan Murali Achary, Malireddy K. Reddy

**Affiliations:** Crop Improvement Group, International Centre for Genetic Engineering and Biotechnology, New Delhi, 110067 India

**Keywords:** Agricultural genetics, Molecular engineering in plants, Plant sciences, Plant biotechnology, Plant molecular biology

## Abstract

Enhancing crop productivity and their nutritional quality are the key components and primary focus of crop improvement strategy for fulfilling future food demand and improving human health. Grain filling and endosperm development are the key determinants of grain yield and nutritional quality. *GRAIN WIDTH and WEIGHT2* (*GW2*) gene encodes a RING-type E3 ubiquitin ligase and determines the grain weight in cereal crops. Here we report *GW2* knockout (KO) mutants in Indica (var. MTU1010) through CRISPR/Cas9 genome editing. The endosperm of *GW2*-KO mutant seed displays a thick aleurone layer with enhanced grain protein content. Further the loss of function of *OsGW2* results in improved accumulation of essential dietary minerals (Fe, Zn, K, P, Ca) in the endosperm of rice grain. Additionally, the mutants displayed an early growth vigour phenotype with an improved root and shoot architecture. The hull morphology of *GW2*-KO lines also showed improved, grain filling thereby promoting larger grain architecture. Together, our findings indicate that *GW2* may serve as a key regulator of improved grain architecture, grain nutritional quality and an important modulator of plant morphology. The study offers a strategy for the development of improved rice cultivars with enriched nutritional quality and its possible implementation in other cereals as well.

## Introduction

Improving crop productivity and grain nutritional quality are the recent focus of crop improvement programme. Plant yield is highly influenced by genetic and environmental factors. Grain yield is an intricate trait of three quantitative complexions of tiller numbers, grain numbers per panicle, and grain weight. The key QTLs of grain architecture have been explored and utilized in modern cultivars for their impact on yield and market values. For instance, *qSW5/GW5* is a major QTL identified to control the grain width and grain weight of rice by regulating cell division during seed development^1^. Similarly, other important genes and QTLs including *GW8*, *GL7/GW7*, *GS5*, *Gn1a*, *TGW6*, *DEP1* and *SPL14* controls wide phenotypic variations in seed shape including grain width, grain weight and grain length, and their molecular mechanism have been well characterized in different rice germplasm^2–8^.

The endosperm is the chief edible part of cereal grains and a major source of human food. Malnutrition and diseases are often linked with poor protein quality and lack of vitamins and micronutrients in the diet and nearly one-third of the world population currently suffers from nutrition-related diseases^9^. The endosperm of rice grain is mainly composed of starchy endosperm and is covered with an aleurone layer. Rice aleurone layer stores lipids, proteins, vitamins, and essential minerals, and constitutes the most important nutritious component of cereal grains. The grain proteins of the major cereals like rice, wheat, maize and barley—contribute an important part of total protein in human food^10^. During the grain filling stage photosynthates, nutrients and other minerals are channelled through the dorsal vascular bundle into developing caryopsis, nucellar projection and finally to endosperm transfer tissue^11^. Through the functional genomic approaches, several genes and QTLs have been identified for their role to control grain architecture. However, only a few genetic controlling mechanisms are so far known for the regulation of grain filling in rice. *GRAIN INCOMPLETE FILLING 1* (*GIF1*) encodes for cell-wall invertase, expressed in the ovular vascular trace and has been identified to take part in carbon partitioning and grain filling^12^. *OsNF-YB* is an aleurone layer specific gene that regulates grain filling and endosperm development by networking with *ERF115*^13^. Also, reports suggest that the *MADS29* gene is highly expressed in the nucellar projection and nucellus tissue and participate in grain filling by degradation of the maternal tissues^14^. Mutation in *NAKED ENDOSPERM* (*NKD*) and *SUPERNUMERARY ALEURONE LAYER1* (*SAL1*) genes accelerate the deposition of endosperms with multiple aleurone cell layers in maize^15,16^. Similar findings have also been reported in the RNAi *RICE SEED B‐ZIPPER 1* (*RISBZ1*) and *RICE PROLAMIN BOX BINDING FACTOR* (*RPBF*) suppressed rice lines^17^. Even though the mutants with multicell aleurone were identified in the germplasm, none of these mutants could be adopted in crop breeding program due to severe defects in grain filling^18^. The recent report reveals that, *REPRESSOR OF SILENCING 1* (*ROS1*) a DNA demethylase promotes aleurone layer thickness and consequently improves the nutritional quality of rice grain^18^. Similarly, rice *thick aleurone 1* (*TA1*) encodes a mitochondrion-targeted protein OsmtSSB1, mutation in *TA1* locus resulted in increased number of aleurone layers and subsequently increase the grain nutritional quality^19^. On the other hand, *DEK1 DEFECTIVE KERNEL1*(*DEK1*) encodes for a plasma membrane integral protein has been identified which is essential for aleurone cell fate specification in maize^20^. Similarly, *CRINKLY4* (*CR4*) identified as a positive regulator of aleurone development encodes for a receptor-like kinase in maize and the cr4 mutants showed irregular patches that lack aleurone layer^21^. The maize *disorganized aleurone layer* mutants (*dil1*, *dil2*) showed a lack of control of the mitotic division plane in the aleurone layer and the cells displayed irregular arrangement of shape and size^22^. A barley mutant *elo2* was also identified which showed disorganization and irregular cellular arrangement in the aleurone layer^23^. The above studies highlight the significance of aleurone in grain filling, and probably too in grain yield and grain nutritional quality.

Rice *GRAIN WIDTH and WEIGHT2* (*OsGW2*), fine mapped from a major QTL identified on the short arm of chromosome 2, is responsible for grain width and weight in rice. *OsGW2* takes part in the ubiquitin–proteasome pathway and regulates cell division of husks during seed development^24^. From protein interaction studies, it was revealed that functional GW2 protein directly interacts with EXPANSIN-like1 (EXPLA1) protein and inactivates it by ubiquitination resulting in short grain size^25^. A SNP in *OsGW2* resulted in premature translational termination of GW2 protein, resulting in enhanced grain width and weight^24^. The orthologs in maize (*ZmGW2-CHR4* and *ZmGW2-CHR5*) also regulate the variation in kernel size and weight^26^. A homolog of *DA2* in wheat (*TaGW2*) has been identified, having a similar function to control kernel size and maturity^27^. In *Arabidopsis*, the *RING-type E3 ligase DA2* negatively controls seed size and its loss of function resulted in a large seed size^28^. In addition to controlling seed size, the other family members of RING-type E3 ligase participates in several developmental and physiological processes in plants including seed germination, dormancy, controlling flowering time, root growth, chloroplast development, self-incompatibility, overall plant growth and also abiotic stress responses towards salinity, drought and temperature stress by regulating or altering the functions of verities of cellular regulatory polypeptides and enzymes^29^. However, a detailed study is necessary to understand the involvement of E3 ligases in resulting other agronomic important traits apart from its role in controlling grain architecture thus enabling its utilization in the future crop improvement programme. The genome editing methods including TALENs, CRISPR-Cas9, base editing and prime editing technology have quickly become the ideal tool for genetic manipulation for characterizing gene function and dissecting complex genetic networks. Many of the genes have been edited by using any of the above methods for improving crop yield, disease resistance, nutritional quality, abiotic stress tolerance^30,31^. Genes associated with agronomically important traits have been characterized mostly in the japonica background^32^. Indica cultivars occupy about 80% of the world’s rice cultivated area. Hence it is necessary to harness the advantages of cutting-edge biotechnology and creates novel and useful genetic variations in the indica germplasm for future crop improvement program. *GW2* locus has been characterized in different rice backgrounds through CRISPR/Cas9 knockout approach^33–35^. The above studies suggest the key role of *GW2* in controlling grain size and weight as previously reported by Song et al^24^. However, the effect of the mutation in the *GW2* on grain nutritional quality was least explored. In this study, we developed *OsGW2* knockout mutants using CRISPR/Cas9 genome editing in indica rice cultivar MTU1010 and functionally characterized to understand its key role in seed development, grain nutritional quality, and also overall plant phenotype.

## Results

### Preparation of Cas9-sgRNA vector system and Generation of *GW2*-KO mutant lines

**Figure 1:**
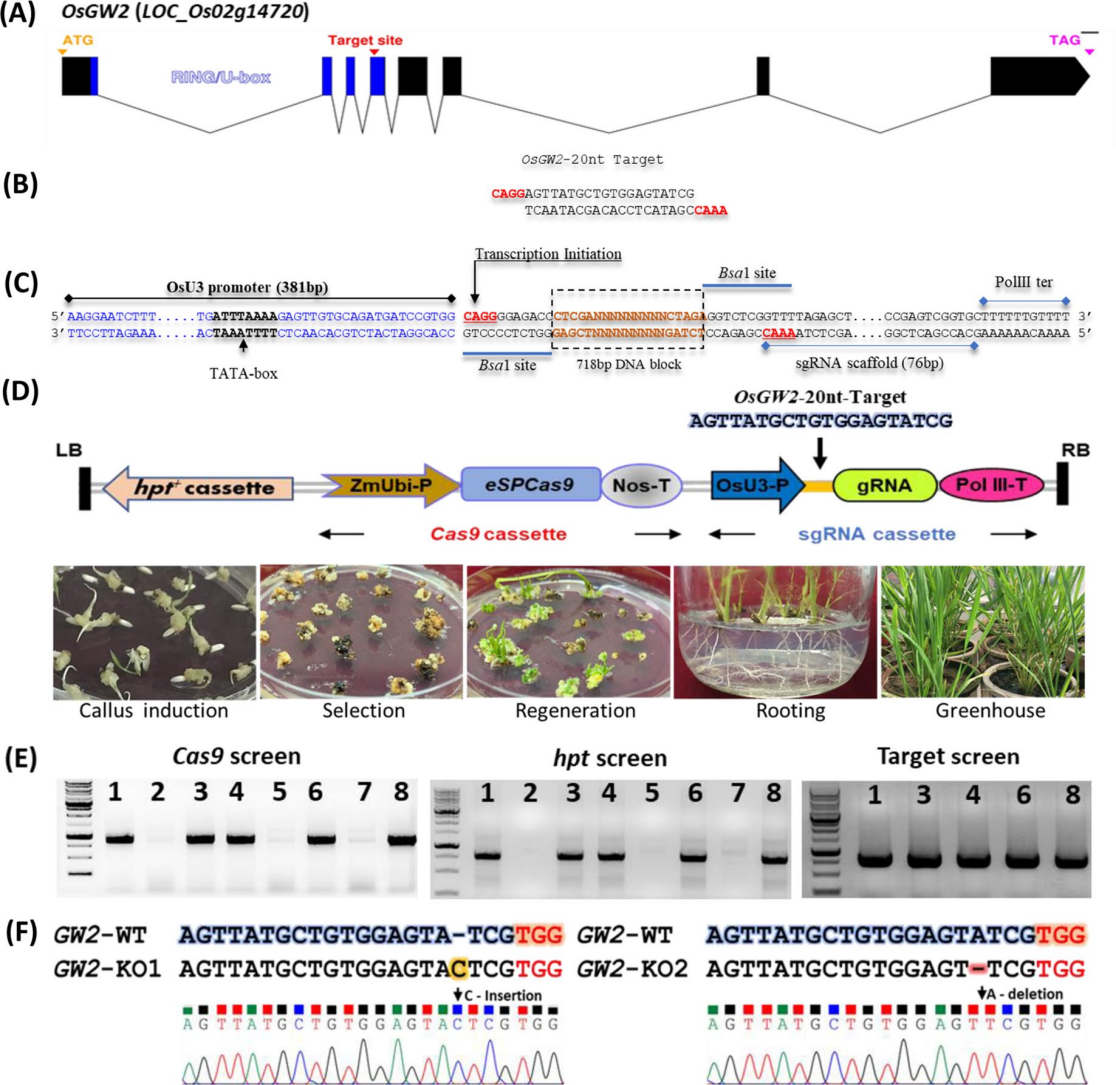
Structural organization of *OsGW2*, *eSpCas9* expression cassette and mutation analysis. (**A**) Rice *GW2* gene consisting of 8 exons interrupted by 7 introns and RING-U box domain highlighted blue. The structural organization of *OsGW2* gene was graphically represented using exon–intron graph maker (http://wormweb.org/exonintron). The 20nt-sgRNA target site was selected at RING/U box domain. (**B**) Specific 20-nt sgRNA target sequence along with *Bsa*I cloning sites. (**C**) sgRNA expression cassette showing rice U3 promoter and polIII terminator consisting two *Bsa*I restriction sites separated by a 718 bp DNA block. (**D**) T-DNA border having genome editing tools (*eSPCas9* and sgRNA) in pMDC99 vector. The lower figure (**D**) represents different stages of plant development following tissue culture methods. (E) PCR confirmation of T0 plants showing presence of *Cas9* (887 bp) and *hpt* (954 bp) genes. The last figure (**E**) showing PCR amplification of 746 bp flanked sequence including the 20nt target region from the *Cas9* positive plants for identification of INDEL mutations. (**F**) Genotyping confirmation of *GW2*-KO mutant lines showing C-insertion (*GW2*-KO1) and A-deletion (*GW*-KO2) mutation generated by *Cas9* nuclease. The image was created by chromatogram viewer Chromas 2.6.6 software (http://technelysium.com.au/wp/chromas/).

The 20-nt sgRNA highly specific to the target site within *GW2* genomic sequence was chosen in the fourth exon of U-box domain, located at 1786 bp downstream of the start codon (ATG) or at 321 bp downstream of the *GW2* coding sequence of rice. The *eSpCas9* expression cassette (ZmUbiP- *eSpCas9*-nosT) and OsU3-gRNA (OsU3-gRNA-PolIIIT) were cloned together into pMDC99 binary vector following multi gateway LR cloning method (Fig. 1D). The *Agrobacterium* strain EHA105 carrying pMDC99-CRISPR/Cas9-U3sgRNA expression cassette was transformed into indica rice MTU1010 cultivar. Following tissue culture, a total of eight independent putative rice lines were generated. Five lines (L1, L3, L4, L6 and L8) were positive for the integration of T-DNA which were confirmed by PCR using *hpt* and *Cas9* gene specific primers, listed in Supplementary Table S1 (Fig. 1E). To identify insertion-deletions (INDELs) in the *Cas9* positive plant, a set of gene specific primers were designed up and downstream of the 20nt-*GW2* target sequence (Supplementary Table S1). A746bp flanked sequence including the 20nt target area was amplified from the putative *Cas9* positive plants and sequenced using internal sequencing forward primer (Fig. 1E, Supplementary Table S1). All *Cas9* positive plants were Sanger sequenced to confirm different INDEL mutation frequencies and types. Among the *Cas9* positive T0 plants, we identified a biallelic mutation with single nucleotide C-insertion in the L1 plant, subsequently referred to as *GW2*-KO1 (Fig. 1F). We genotyped over 189 individual progenies in the T1 generation of all *Cas9* positive lines. Additionally, in the T1 progeny of *Cas9* positive plant L6, a single A-deletion biallelic mutation was also identified and subsequently the mutation was referred to as *GW2*-KO2 (Fig. 1F). In addition to the above mutations, we did not notice any additional mutations in the T1 progeny of L3, L4 and L8. The editing efficiency of *Cas9* gene is chiefly dependent on the gRNA sequence and its secondary structure. The effect of INDEL mutations on the full-length protein frame were predicted using Expasy bioinformatic tool. Both the C-insertion and A-deletion mutation end up with truncated GW2 peptides (Supplementary Fig. S9). Seeds from *GW2*-KO1 and *GW2*-KO2 homozygous mutants were harvested at maturity, and T2 seedlings were raised. The T-DNA free *GW2*-KO mutant lines with above mutation types were identified in the T2 progeny. The T3 *GW2*-KO mutant plants were advanced to next the generation. The above mutations were inherited in the next generation progeny which was confirmed through Sanger sequencing. All the agronomic and morphological parameters were measured in the T-DNA free *GW2*-KO homozygous mutant lines.

### Grain aleurone phenotyping and quantification of grain nutritional content

**Figure 2:**
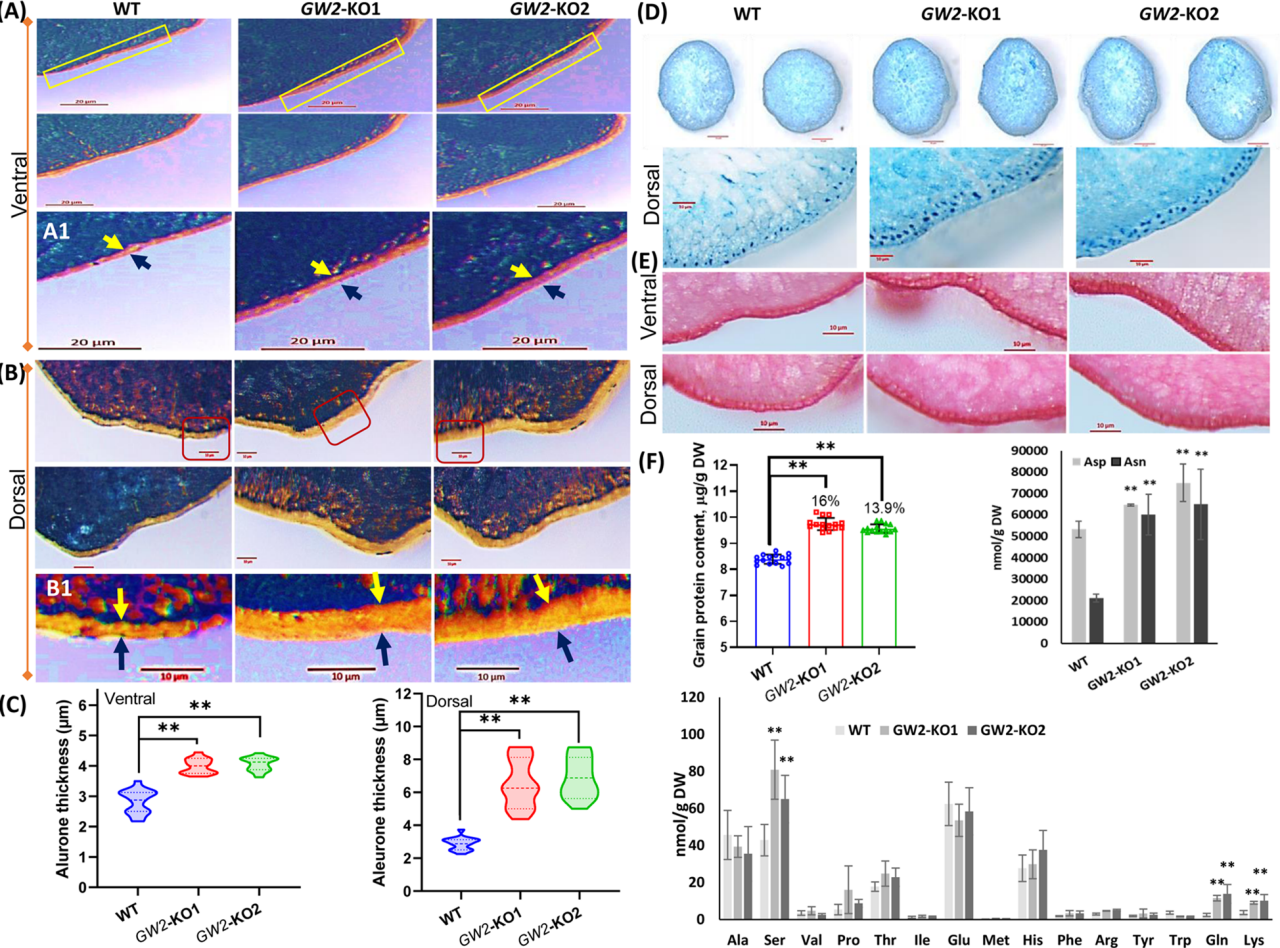
Characterization aleurone density and grain protein content of GW2-KO mutant. Transversally sectioned (1–2 mm size) dehusked mature rice grains from WT and *GW2*-KO lines, stained with iodine solution for visualization aleurone morphology. The ventrolateral (**A**) and dorsolateral (**B**) section showing aleurone layer morphology of the WT and *GW2*-KO lines. Magnified visualization of ventrolateral (A1) and dorsolateral (B1) sections of rectangular box from (**A**, **B**). (**C**) The aleurone layer density of *GW2*-KO lines significantly *p* ≤ 0.01 (**) improved both in ventral and dorsal surface compared to WT seed. Arrowheads indicate the aleurone layer thickness. (**D**) The transversally sectioned (1–2 mm size) rice grains from WT and *GW2*-KO lines stained in Bradford reagent. The *GW2*-KO mutants showed high intense dark blue color compared to WT seed. The lower figure represents the closer view of aleurone layer of WT and *GW2*-KO lines. The aleurone layer of *GW2*-KO exhibits more protein bodies compared to WT. The scale bar represents 10 or 20 µm respectively. Data ± SD (n = 15). (**E**) Transversally sectioned of WT and *GW*-KO mature grains, stained in 0.05% (w/v) Safranin. Both WT and *GW*-KO showed single aleurone cell layer morphologies. The GW2-KO mutants exhibited larger cell size in the aleurone layer compare to WT seed. (**F**) The grain protein content of *GW2*-KO mutants was substantially increased (*p* ≤ 0.01) compared to WT (n = 15). (G-H) The UPLC-MS/MS analysis of *GW2*-KO seeds showed significantly *p* ≤ 0.01 (**) higher free amino acid accumulation (Ser, Gln, Lys, Asp and Asn) compared to WT. Data ± SD (n = 6).

We checked the grain nutrition quality of *GW2*-KO mutants. Interestingly, both the mutants showed improved aleurone layer morphology. Iodine makes a complex with starchy endosperm and turns into deep orange-brown color except for the aleurone layer. From the microscopic observation, we found that the aleurone layer thickness substantially (*p* ≤ 0.01) improved in *GW2*-KO lines on both ventral (40–44%) and dorsal (126–137%) surfaces compared to WT seed (Fig. 2A–C). Compared to the ventral side the aleurone thickness was significantly more in the dorsal side of rice grain. Further, we confirmed that there was no significant difference in the outer pericarp layer thickness in both WT and *GW2*-KO lines. Also, we did not notice any significant increase in the additional aleurone layers in the *GW2*-KO mutant lines (Fig. 2E). However, the *GW2*-KO lines displayed improved aleurone layer cell size compared to WT (Fig. 2E). The total grain protein content (GPC) of *GW2*-KO substantially (*p* ≤ 0.01) increased (13–16%) with respect to WT plant (Fig. 2D, F). The protein quantitative data also support the histochemical stain of rice grain with Bradford reagent (Fig. 2D). The closer microscopic observation further revealed that *GW2*-KO lines have more protein bodies and are predominantly located in the endosperm and aleurone tissue of rice grain (Fig. 2D). Further, we analysed the level of amino acid following UPLC-MS/MS method. Compare to the corresponding WT seed, the free amino acids including Ser, Gln, Lys, Asp and Asn were significantly (*p* ≤ 0.01) more in *GW2*-KO lines (Fig. 2F). The findings indicate that *GW2* is a potential allele in breeding to accomplish grain yield as well as nutritional quality in rice.

**Figure 3:**
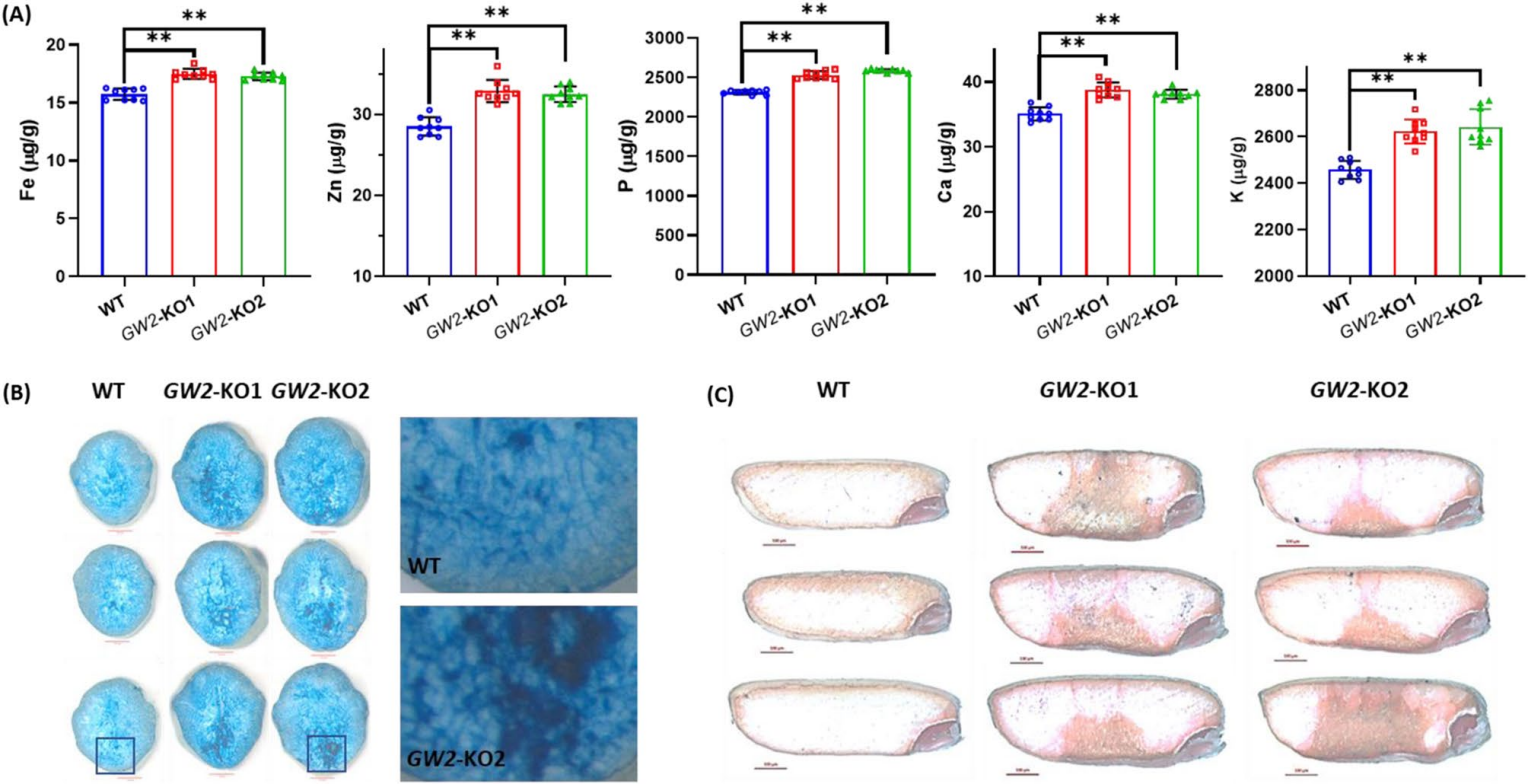
Assessment and distribution of grain mineral content. (**A**) ICP-MS quantification mineral content (Fe, Zn, P, Ca and K) in the mature dehusked rice seeds. The seed iron (9–11%), zinc (13–15%), phosphorus (9–11%), calcium (8–10%) and potassium (6–7%) content were significantly *p* ≤ 0.01 (**) increased in the *GW*-KO rice lines. Data ± SD (n = 9). (**B**) The transverse cut section showing variation in the Prussian blue stain intensity among WT and *GW2*-KO lines. The *GW2*-KO lines accumulates improved iron content in the rice endosperm compared to WT seed. Scale bar 50 µm. (**C**) The dithizone stained longitudinally cross section of half seed form WT and *GW*-KO lines showing different degrees of zinc deposition. The *GW*-KO lines accumulates higher amount of zinc metal in the endosperm tissue compared to WT seed. Scale bar 100 µm. Microscopy was performed using Carl Zeiss stereoscopic zoom microscope (Discovery V8) attached with cooled digital camera.

Rice is one of the important and easily accessible sources of micronutrient minerals for humans. Further, we measured the grain mineral content of WT and *GW2*-KO using inductively coupled plasma mass spectrometry (ICP-MS). Interestingly, the total grain iron (9–11%), zinc (13–15%), phosphorus (9–11%), calcium (8–10%) and potassium (6–7%) content of *GW2*-KO lines were significantly (*p* ≤ 0.01) higher in compared to WT (Fig. 3A). The increased iron content in the *GW2*-KO seed was also confirmed by the Prussian blue biochemical staining method (Fig. 3B). In agreement with the ICP-MS analysis, the *GW2*-KO showed strong blue staining for iron in the endosperm (Fig. 3B). Similarly, the histochemical visualization of dithizone method strongly indicates that *GW2*-KO accumulates higher amount of zinc in the endosperm (Fig. 3C). Further, we identified a unique strong distribution of iron and zinc towards the ventral half of the seed endosperm (Fig. 3B, C). The strong intense stain indicates the presence and distribution of high iron and zinc concentration in the *GW2*-KO seeds. From the above study, we conclude that *OsGW2* locus controls grain nutritional quality, and loss of function improves grain protein content, enhances aleurone layer and increases mineral content in rice seeds. Thus, the study offers a strategy for the development of biofortified rice and such orthologous mutants will probably have enhanced nutritional status in other cereal crops.

### Agronomic performance of *GW2*-KO mutant

**Figure 4:**
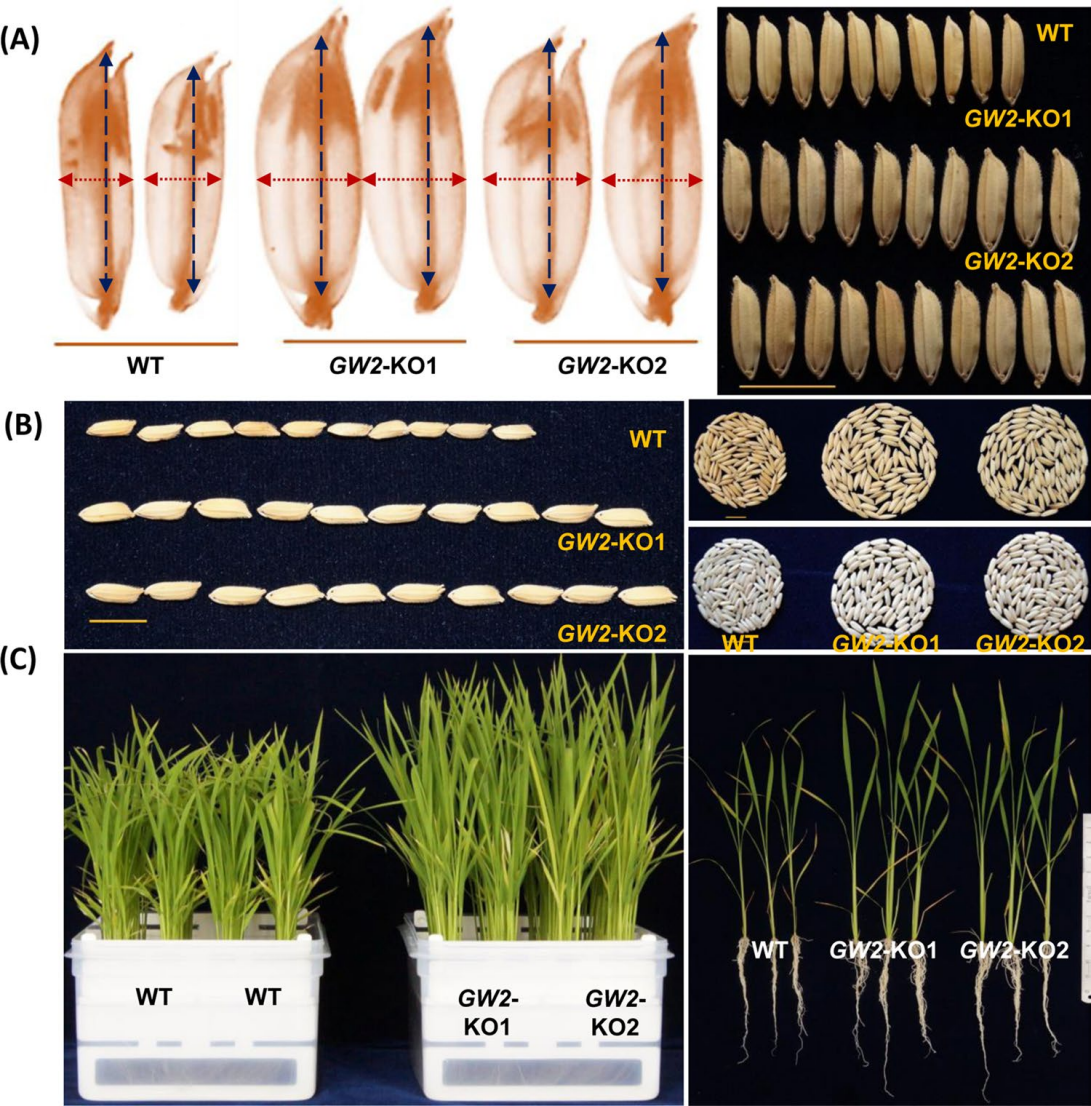
Grain phenotype and agronomic performance of *OsGW2*. *GW2*-KO mutant lines showed improved hull morphology and seed architecture and grain width compared to WT plant (**A**). (**B**) The *GW2*-KO lines exhibited increased seed length and 100 grain volume corresponding to WT line. (**C**) Phenotyping of 30-day old *GW2*-KO lines showing improved root-shoot length and biomass corresponding to WT plant.

The agronomic performance of *GW2*-KO mutants were recorded under field conditions. Before the grain filling stage, the hull size of *GW2*-KO lines significantly improved in both in longitudinally (12–13%) and transverse (66–67%) direction (Fig. 4A, Table 1). The improved hull size accelerates grain filling and channelizes more photosynthetic carbon into shrink tissue resulting in bigger seeds (Fig. 4A). We reported a 42–44% increased width and a 27–32% enhancement in seed length in *GW2*-KO lines (Fig. 4A,B). Similarly, 1000-grain weight was improved substantially (33–34%) however, the number of grains per main panicle was reduced by 22–24% in the *GW2*-KO lines (Table 1). Our result indicates that the functionally active *GW2* allele negatively acts on grain width, length and 1000 grain weight, however positively correlated with the number of grains per panicle in rice. Further, we noticed that the *GW2* locus contributed more toward the grain width (42–44%) compared to grain length (27–32%) (Table 1). Furthermore, we investigated the effects of mutation on the plant phenotype. The *GW2*-KO plant showed improved seedling growth morphology with significantly (*p* ≤ 0.01) improved leaf length (17–19%), root length (11–13%), number of roots per plant (30–36%), shoot biomass (68–69%) and root biomass (57–75%) with respect to WT plants (Fig. 4C, Table 1).

**Table 1:** The hull length and width, grain length and width, plant height, 1000 grain weight increased significantly *p* ≤ 0.01 (**) except grain number per min panicle which is significantly decreased compared to WT. Seedling phenotype of *GW2*-KO lines significantly *p* ≤ 0.01 (**) improved root number root length, root and shoot biomass compared to WT. Data means from independent 15 (± SD) plants.

(A) Agronomic performance of WT and *GW2*-KO lines							
	Hull width (cm)	Hull length (cm)	Plant height (cm)	Grains/main panicle	Grain length (cm)	Grain width (mm)	1000 grain wt (gm)
WT	0.35 ± 0.05	1.2 ± 0.03	97.4 ± 4.2	157 ± 8.3	0.84 ± 0.7	2.4 ± 0.2	23.4 ± 0.2
*GW2*-KO1	0.58 ± 0.07 (+ 66%)**	1.39 ± 0.03 (+ 12.3%)**	100 ± 3.1 (+ 2.6%)**	122.3 ± 8.7 (− 22.1%)**	1.1 ± 0.8 (+ 32.5%)**	3.4 ± 0.3 (+ 44.2%)**	31.5 ± 0.5 (+ 34.6%)**
*GW2*-KO2	0.59 ± 0.07 (+ 67.9%)**	1.4 ± 0.04 (+ 13.4%)**	99.8 ± 1.8 (+ 2.4%)**	118.6 ± 10.1(− 24.4%)**	1.07 ± 0.7 (+ 37.7%)**	3.4 ± 0.3 (+ 42%)**	31.3 ± 0.5 (+ 33.7%)**

(B) Seedling phenotyping of WT and *GW2*-KO lines							
	Main leaf length (cm)	Main root length (cm)	No. of root/plant	Shoot biomass (gm)	Root biomass (gm)		
WT	22.9 ± 1.5	21.8 ± 1	18.7 ± 1	0.73 ± 0.08	0.47 ± 0.03		
*GW2*-KO1	27.5 ± 0.8 [19.9%; **]	24.4 ± 2.3 [11.9%; **]	25.6 ± 3 [36.9%; **]	1.24 ± 0.27 [69.9%; **]	0.83 ± 0.16 [75.3%; **]		
*GW2*-KO2	26.8 ± 1.5 [17.2%; **]	24.8 ± 1.7 [13.7%; **]	24.5 ± 1.5 [30.9%; **]	1.23 ± 0.19 [68.4%; **]	0.75 ± 0.13 [57.4%; **]		

## Discussion

Rice grain yield and quality are important and complex agronomic traits, controlled by several genes and their interactions. Grain architecture is mostly governed by genetic factors. However, the ratio of filled grains is strongly affected by environmental factors. Grain size is a major target of breeding, not only as a component of grain yield but also as a quality trait that determines the market value. The *GW2* and its homoeologs have been experienced during selection, domestication and artificial breeding programme in different crops^36^. *GW2* gene locus encodes for RING-type E3 ubiquitin ligase and has been identified as the major QTL responsible for improving the hull size which promotes grain filling rate and endorses improved grain width and size in rice^24^. However, there is a lack of knowledge on the grain filling rate and nutritional quality with respect to grain size in rice. Genome editing platform has emerged as a cutting-edge molecular tool for manipulating the plant genome in many ways for dissecting gene function and developing improved crop varieties. CRISPR/Cas9 genome editing has been employed to knockout *GW2* locus in different rice background. For example, multiplex gene editing has been used to simultaneously knockout three important genes namely *Grain Width and Weight 2*, *Grain Width 5* (*GW5*), and *Thousand-Grain Weight 6* (*TGW6*) in the NIL-*Grain Size3* LH422 rice background. The homozygous *gw5tgw6* and *gw2gw5tgw6* mutants remarkably increased thousand-grain weight (TGW) than that of wild-type LH422. Additionally, the grain size and TGW of the *gw2gw5tgw6* mutants were significantly larger than the *gw5tgw6*, signifying *GW2* might function independently of *GW5* and *TGW6*^33^. Similarly, Zhou and colleagues employed CRISPR/Cas9 multiplex genome editing to simultaneously edit three yield-related QTLs including *OsGS3*, *OsGW2*, and *OsGN1a* in japonica rice varieties like Jijing809 (J809), Liaojing237 (L237), and Chuan Nong Xiang Jing (CNXJ) for improved grain yield. The biallelic triple mutant (*gs3, gw2 and gn1a*) significantly improved panicle length, grains per panicle, and weight per grain in both J809 and CNXJ varieties. Furthermore, the grain length and width of L237 rice with genotype *gs3gs3gw2gw2GNa1gn1a* significantly increased over WT control. Overall, the finding also highlights the additive effect of genes in triple mutants of J809 and L237 varieties which resulted in 68 and 30% increased yield per panicle^34^. *KEMS39* was identified as a natural mutant in the ‘Koshihikari’ rice background which contains a 67 bp deletion in the 3ʹ splice site of the sixth intron of the *GW2* gene. The mutant showed increased grain size and yield with improved lodging resistance. The above agronomic characteristics were also confirmed in *gw2* knockout mutant generated using CRISPR/Cas9 contains a 7 bp deletion including in the same 3′ splice site^35^. These results suggest that the mutation in *OsGW2* had predicted effects on grain size and weight. However, the effect of the mutation in the *GW2* gene on grain nutritional quality was least explored. Apart from its role in controlling grain architecture, in the present finding we identified the key role of *OsGW2* as regulator of aleurone layer morphology, grain nutritional quality, and overall plant morphology in rice. The triploid rice endosperm directly provides 40% of dietary protein to human. Grain appearance, nutritional value and cooking quality are linked to the composition of protein and starch in the endosperm. Aleurone layer is the main reservoir of micronutrients in rice grain. Our results indicate that *GW2*-KO mutants had substantially improved aleurone layer compared to WT seeds (Fig. 2A–C). A number of genetic factors such as *CRINKLY 4* (*ZmCR4*), *DEFECTIVE KERNEL 1* (*ZmDEK1*), and *SUPERNUMERY ALEURONE LAYER 1* (*ZmSAL1*) control different aspects of aleurone layer. The *SAL1* mutant showed multiple layers of aleurone cells suggesting negative functions of aleurone fate^16^. Interaction studies suggested that the *CR4* and *DEK1* work together in determining the aleurone cell fate however, both are negatively regulated by *SAL1* which encodes for a class-E vacuolar sorting protein^37^. *OsRISBZ1* and *OsRPBF* are the zinc finger transcription factors that function together and enhance the expression of storage proteins of rice endosperm^38^. Transgenic studies suggest that knockdown of *OsRISBZ1* had no effect on aleurone fate however, repression of *OsRPBF* resulted in irregular multi-layered aleurone. While co-repression of *RISBZ1/RPBF* double knockdown line showed improved aleurone consisting of multiple layers of large, disordered cells. Other genes including *thick aleurone 1* (*Zmthk1*) and *extra cell layer 1* (*Zmxcl1*) mutants act as potential negative regulators of aleurone development in maize^39,40^. Although the above genes have been identified to positively or negatively regulate the aleurone layer number, these genetic lines showed various defects in plant development (root, shoot and leaf) and other agronomic traits including grain size, seed setting rate, germination, and viability. In contrast, the *GW2*-KO showed fast germination with improved seedling biomass (Fig. 4C). Reports suggest that plant hormones also influence aleurone fate. Aleurone differentiation at an early stage is affected by phytohormone auxin and cytokinin^37^. Transgene overexpression of *isopentenyl transferase* (*IPT*) under senescence-responsive SAG12 promoter in maize resulted in mosaic aleurone development, indicating that cytokinin has an inhibitory consequence on aleurone fate^41^. On the other hand, treatment with N-1-naphthylphthalamic acid (NPA), an auxin transport inhibitor resulted in development of multiple layered aleurone in maize. The immune-histochemical detection of indole acetic acid disclosed that the NPA treatment caused accumulation of auxin in the endosperm periphery. Also, evidence of an increased level of *ZmPIN1* expression in the NPA treated plant. The study suggests auxin has positive association with aleurone development, promotes aleurone cell fate and resulted multilayered aleurone in NPA treated maize plant^42^.

Rice aleurone is the core contributor of a spectrum of micronutrients including lipid, protein, minerals, vitamins, fibers and antioxidants. Hence, we hypothesize that the improved aleurone layer may contribute to increased grain protein content and mineral nutrient in the *GW2*-KO mutants. We quantified the total grain protein content as well as profiled the free amino acid in rice grain. Interestingly, the *GW2*-KO seeds accumulate substantially more (12–14%) total grain protein content compared to the WT seeds (Fig. 2D). The histochemical staining of *GW2*-KO seeds further indicated the presence of more protein bodies both in the endosperm as well as aleurone tissue (Fig. 2D). Further, the substantial increase in the protein content of *GW2*-KO can be correlated with higher accumulation (Ser, Gln, Lys, Asp and Asn) free amino acids in the grain (Fig. 2D, F). Digestibility and grain protein quality of rice is high due to the presence of a high amount of lysin as compared to other cereals^43^. Therefore, enriching the GPC in rice is one of the major focus in agriculture biotechnology and breeding science. Rice grain nutritional quality is a complex genetic trait that has been linked with many QTLs and genes. GPC is a key factor in determining nutritional value and the genetic controlling mechanisms associated with GPC remain unclear. It has been shown that overexpression of *aspartate aminotransferase* (*AST*) leads to an increase in the total amino acid pool by 2.0–5.4% and grain protein by 22.2% in rice^44^. Similarly, qPC1 is a major QTL that has been identified as a positive regulator of GPC in rice. The *qPC1* encodes for a putative amino acid transporter *OsAAP6*, which significantly contributes to the accumulation of glutelins, prolamins, globulins, albumins and starch in rice seed^45^. The map-based cloning after crossing indica and japonica cultivars, revealed that a major QTL (qGPC-10) that encodes for a glutelin type-A2 is involved in the regulation of grain protein content in rice. The study further revealed that a single SNP present in the promoter region of *GluA2* leads to high transcript expression and causes increased GPC in the indica rice cultivar^46^. Generally, cereal proteins contain a low level of lysine (1.5–4.5), tryptophan (0.8–2.0), and threonine (2.7–3.9) as against 5.5%. 1% and 4.0% respectively recommended by WHO. We reported 2.4–2.7 fold (142–173%) increased lysine content in the *GW*-KO lines compared to WT. The increased lysine content positively correlated with the improved grain protein content in *GW*-KO lines. It is well established that the digestibility of rice bran protein is higher (94.8%) in comparison to rice endosperm protein (90.8%) and soy protein (91.7%) considered the same as that of milk protein casein. Therefore, rice bran protein appears to be a promising protein source with high biological value and good digestibility^47^. Rice *GRAIN INCOMPLETE FILLING 1* (*OsGIF1*) encoding a cell-wall invertase, expression is required for carbon partitioning during early grain-filling and is negatively regulated by *GW2*^48^. Overexpression of *OsGIF*1 in rice by its native promoter resulted increase in grain production^12^. In agreement with the previous finding our reports suggest that *OsGW2* locus is perhaps involved in the translocation free amino in the rice grain through modulation of transporters including *GIF*1. Recently it was demonstrated that the protein content of hexaploid common wheat (*Triticum aestivum* L.) was significantly increased in the mutant lines that lacked two or three (*TaGW2-A1*, *-B1* and *-D*) homoeologs of *TaGW2*^49^. Further, our study revealed that *GW2*-KO accumulated substantially higher essential minerals including iron, zinc, calcium, phosphorus, and potassium in the rice endosperm (Fig. 3). The recent report suggests that vascular transporters are vital for the distribution and loading of iron in the rice grain. The *Yellow Stripe-Like* plasma membrane transporter (*OsYSL2*, *OsYSL9* and *OsYSL15*) expressed predominantly in the outer layer and surrounding cells of endosperm, plays an important role in the translocation, distribution and accumulation of Fe, Mn in rice endosperm^50^. Similarly, other membrane transporters *VIT1* and *VIT2* in rice also appear to participate in long-distance mobilization of zinc and Fe between flag leaves (source) and seeds (sink organs) via the modulation of flag leaf Zn and Fe buffering pool^51^. Further studies suggest that sugar transporters expressed in the aleurone layer and participate in sugar loading and grain filling. For example, rice hexose transporters both *monosaccharide transporter 4* (*OsMST4*) and *OsMST6* are expressed in the grain tissue including nucellar epidermis, nucellar projection, dorsal vascular bundle, and aleurone layer^52,53^. Similarly, *OsCINs* and *OsMSTs* are participating in the loading of monosaccharides into the rice endosperm^53,54^. The rice *NUCLEAR FACTOR Y B1* an aleurone-specific transcription factor triggers the expression of *OsSUT1*, *OsSUT3*, and *OsSUT4* sucrose transporters and facilitate grain filling^11,55^. *OsSWEET11* and *OsSWEET15* are also reported during sugar loading into the endosperm tissue^56,57^. Our findings suggest that *GW2* controls grain nutrition and the null mutants accumulate more iron and zinc, mainly distributed in the ventral half of rice endosperm. Additional studies are required to look into the specific role of transporters which might be directly involved in the loading and mobilization of minerals to the seed endosperm and their regulation by the ubiquitin–proteasome pathway.

Further we notice, the loss of function of *OsGW2* locus enhances the spikelet hull size both in length and width (Fig. 1A; Table 1). The enlarged hull size sequentially accelerates more grain filling and diverts photosynthates into the hull resulting increased grain width and weight. The present finding is concomitant with experimental evidence from the previous reports^24^. Recent studies from the yeast two-hybrid and in vitro pull-down assays revealed that GW2 protein strongly interacts with expansin-like 1 (EXPLA1) of the hull. EXPLA1 is a cell wall-loosening protein that increases cell growth of rice spikelet hull. In vitro studies further confirmed that EXPLA1 was ubiquitinated by GW2 at lysine 279 and caused degradation of EXPLA1 through its E3 ubiquitin ligase activity and negatively regulated the seed size in rice^25^. The *GW2*-KO lines showed improved grain length (27–32%), grain width (42–44%) and 1000 grain weight (33–34%) however the grain number per panicle was reduced (Table 1) compared to WT rice. Studies have been suggested that the *GW2* locus not only control grain width but also grain length in rice. Zhou et al. and co-workers have identified the CRISPR/Cas9 generated *gw2* knockout mutant resulted in a significant increase in the grain length in the *Oryza sativa* japonica var. Jijing809^34^. Similarly, a recent study also showed that *GW2*-KO mutant developed in Nipponbare cultivars using CRISPR/Cas9 had significantly longer rice seeds^35^. Song et al. also reported that the grain length of near isogenic line, NIL-*GW2* was slightly more^24^. From the various studies, it was evident that rice *GW2* homologs also exist in other crops including maize, wheat and sorghum^26,27^. Furthermore, it was also noticed that simultaneous mutation in *TaGW2* homoeologs (*TaGW2-A1*, *-B1* and *-D1*) significantly increased the grain length architecture in common hexaploidy wheat (*Triticum aestivum* L., AABBDD)^58^. Hence, our findings are consistent with the earlier studies indicating that the functional *OsGW2* gene is a negative regulator grain width, weight and length^24,34,35,58^. Further, we reported that *GW2*-KO showed significantly increased root-shoot length and biomass compared to WT plant (Fig. 4C, Table 1). The involvement of E3 Ubiquitin Ligases in plant development and the hormone signalling processes were well documented both in dicots and monocots^29^. Recently, using transcriptome analysis revealed about 1426 differentially expressed genes in an *OsGW2*RNAi transgenic line^59^. Among these, 115 genes were identified with specific functions including seed, leaf, root, and shoot development, cell cycle regulation and hormone signaling pathway. Further, they identified that most of the auxin-responsive genes (*OsMGH3*, *OsMADS29*, *OsRAA1*, and *OsIAA9*) and the cytokinin and brassinosteroid-related genes were upregulated in the *OsGW2* knockdown plants. Considering the above facts, the improved root and shoot length of *GW*-KO lines is perhaps due to the possible involvement of *OsGW2* in the modulation of phytohormone pathway genes in the rice plants. Overall, our study highlights the pleiotropic role of *OsGW2* not only in regulating the grain weight also in modulating plant architecture and grain nutrition.

In conclusion, the study pointed out the role of *GW2*, acting as a key regulator for improved grain architecture, aleurone morphology, and modulator of grain nutritional quality in rice. *GW2* thus emerges as a new genetic determinant and an ideal genetic resource for the future breeding program for the development of improved cultivar. The novel null allele with enhanced trait values can be directly deployed into commercial cultivation systems or as donor parents in breeding programmes for developing biofortified crops for future food and nutritional security. Further, a comprehensive and integrated research strategy is required to pyramid more favourable genetic determinants through multiplex genome editing system in order to achieve rapid generation of high-yielding nutritionally improved rice cultivars for future food security.

## Materials and methods

### Plant materials and growth conditions

Most of the chemicals were purchased from Sigma Chemical Corporation, Ltd. (St. Louis, MO). Primers used in this study were synthesized by Integrated DNA Technologies (Leuven, Belgium). LR clonase, purchased from Thermo Fisher Scientific Corporation, USA. Restriction enzymes were obtained from New England Biolabs, MA. MTU1010 rice seeds were obtained from ICAR-IIRR, Hyderabad. Plant phenotyping studies were conducted in the paddy field at ICGEB, New Delhi and greenhouse conditions (14/10 h light/dark cycle illumination at 370 µE m^−2^ S^−1^ and 27 ± 1 °C with 70% relative humidity) with proper biosafety levels. The WT referred to the untransformed parent MTU1010 rice genotype.

### Preparation *Cas9* and sgRNA expression cassettes and vector construction

The rice codon-optimized enhanced *SpCas9* (*eSpCas9*) fused with the N-terminal nuclear localization signals and C-terminal nucleoplasmin signal sequence was synthesized by GeneArt (ThermoScientific, USA) (Supplementary Figs. S1, S2). To minimise the off-target editing, three amino acid substitutions were introduced at 887/K, 1042/K and 1099/R substituted with A to improve a high level of on-target cleavage specificity which is broadly useful for genome editing applications (Supplementary Fig. S1). Similarly, the sgRNA expression cassette was prepared under the regulation of rice U3 promoter also separately synthesized by GeneArt gene synthesis service (Supplementary Fig. S3). The optimized *eSpCas9* was sub-cloned into a Gateway compatible entry vector EV-1 (pL12R34-Amp) in between the maize polyubiquitin1 promoter (ZmUbi1P) and nopaline synthase gene (nos) terminator for the high level of expression in the rice (Fig. 1D, Supplementary Figs. S4–S7). Similarly, the OsU3-gRNA expression cassette was cloned into the Gateway compatible entry vector1 (EV-1; pL12R34-Ap) (Fig. 1C, Supplementary Figs. S6, S8). The *Bsa*1 site was introduced both along in the OsU3-gRNA expression cassette for cloning of 20nt target site sequence into 3’GTCC (adjacent to U3promoter) and 5’GTTT (adjacent to sgRNA) overhang sequences (Fig. 1C, Supplementary Fig. S3). A 718 bp gene block was introduced between the two *Bsa*1 sites of OsU3-gRNA expression cassettes. These gene blocks will be released during restriction digestion of EV1 plasmids by *Bsa*1 enzyme to ensure the complete digestion of EV1 plasmid DNA by *Bsa*1 enzyme (Fig. 1C, Supplementary Figs. S3, S8). The Supplementary Figures S6, S7 and S8 were created by SnapGene 5.1.5 software (from Insightful Science; available at https://www.snapgene.com).

To generate *GW2*-KO mutant, we design single sgRNA targeting to ring U box domain of *OsGW2* locus (Fig. 1A). The specific target site was chosen in the fourth exon of U-box domain, located at 1786 bp downstream of the start codon (ATG) within *GW2* genomic sequence. The CRISPR-direct (https://crispr.dbcls.jp/) and CHOPCHOP v2 (https://chopchop.cbu.uib.no/) in silico analysis were performed to design a specific target sequence of *GW2* with an accepted range of GC content (40–70%) against the indica rice genome (*ASM465v1*)^60^. Unique pair of 20nt oligos along with *Bsa*I cloning sites were chemically synthesized (IDT, Inc.) introducing *Bsa*I cloning sites CAGG and CAAA in the 5’ end of forward and reverse primer respectively (Fig. 1B, Supplementary Table S1). The four-nucleotide overhang 5’CAGG sequence in the forward primer used for cloning of oligo duplex target sequence in the *Bsa*I digestion site of EV1-U3-sgRNA vector which has a transcription start site with ‘A’ nucleotide in the CAGG motif to ensure the high level of sgRNA expression by the rice U3 promoter (Fig. 1C). The DNA oligo-duplex was prepared to mix forward and reverse target sequence (100 µM each) and incubate 42 °C for 30 s followed by 95 °C for 5 min and cool down to 25 °C at 0.1 °C/s resulting in DNA oligo-duplex with 4-nt overhangs at both 5’ ends as shown in Fig. 1B. The OsU3-gRNA expression cassette was digested with *Bsa*1 enzyme and the DNA oligo-duplex were then ligated into *Bsa*I-digested vectors following the protocol^61^. The expression clones obtained were confirmed by sequencing.

The *eSpCas9* (ZmUbiP- *eSpCas9*-nosT) and OsU3-sgRNA (OsU3-sgRNA-PolIIIT) expression cassettes from the entry vectors were sequentially cloned into a gateway compatible plant transformation destination vector (pMDC99) using a multiround LR recombinase gateway method (Invitrogen, USA) (Fig. 1D). Briefly, the EV1-*eSpCas9* expression cassette was initially cloned into pMDC99 vector followed by a second LR recombination reaction with empty EV2 vector and finally with EV1-OsU3-gRNA (OsU3-sgRNA-PolIIIT) expression cassette. The resultant recombinant expression clones obtained after LR recombination were confirmed by sequencing. After transformation into *Agrobacterium* (EHA105), the construct was subsequently used for rice transformation for the development of knockout lines.

### *Agrobacterium*-mediated rice transformation in MTU1010 cultivar

*Agrobacterium* mediated rice transformation was carried out following the method of Manimaran et al. with few modifications^62^. Mature seeds of the indica rice cultivar MTU1010 were used for the stable transformation. In brief, dehusked seeds were sterilized with 70% ethanol for 2 min and subsequently with 2% sodium hypochlorite for 20 min. The seeds were then rinsed five times with sterile water and dried over sterile filter paper. The seeds were transferred on callus induction solid medium (MS salts and vitamins 4.4 g, D-maltose-30 g, casein hydrolysate-0.4 g, L-proline-0.7 g, 2,4-D 2.5 mg/l, sorbitol 0.5 g and gelrite 4 g, pH 5.8) at 27 °C under dark for 2 weeks. Actively growing calli were subculture for 1 week at 28 °C under the dark. Cultured *Agrobacterium* cells were collected and resuspended in MS liquid medium containing 100 μM acetosyringone (OD_600_ = 0.6). Rice calli were immersed in the *Agrobacterium* suspension for 30 min with constant shaking at 50 rpm/min and were dried on sterilized filter paper and co-cultured on a solid medium (MS salts and vitamins 4.4 g, d-glucose 10 g, sucrose 20 g, L-proline 0.5 g, 2,4-D 1.5 mg, acetosyringone 200 µM and gelrite 4 g, pH 5.2) at 25 °C under the dark in the growth chamber for 2 days. The infected calli were washed five times with sterile water containing augmentin 600 mg/l to remove excessive *Agrobacterium*. The calli were transferred into selection media (MS salts and vitamins 4.4 g, sucrose-30 g/l, Casein hydrolysate-0.4 g/l, proline 0.7 g, sorbitol 1 g/l, augmentin 600 mg/l, 50 mg/l hygromycin, 2,4-D 3 mg, gelrite 4 g, pH-5.8). After four rounds of selection, the secondary calli were transferred to pre-regeneration media for 20 days in the dark further transferred into regeneration media (MS salts and vitamins 4.4 g, sucrose 30 g, proline 0.5 g, sorbitol 0.5 g, BAP 2 mg, kinetin 1 mg, NAA 0.5 mg, augmentin 600 mg/l, hygromycin 30 mg/l gelrite 4 g, pH-5.8) in light until the formation of young planets. After 3–4 weeks the regenerated plantlets were transferred into rooting media (MS salts and vitamins 2.2 g, D-sucrose-15 g, gelrite 4 g, pH-5.8) for proper root growth, subsequently into soil pots maintained at 70–80% humidity.

### Identification of INDEL mutation

Genomic DNA was isolated from the leaves of putative tissue culture generated rice plants, following the sodium dodecyl sulfate method. PCR amplifications were carried out using *Cas9*, *hptII* and *OsGW2* gene specific primers using 150 ng genomic DNA as a templet. The ZmUbi promoter forward and *Cas9* gene specific reverse primer were used and 887 bp PCR product was amplified to confirm the presence of *Cas9* expression cassette (ZmUbiP-*Cas9*-NosT). Similar, internal *hptII* gene specific forward and reverse primers were used for amplification of 954 bp DNA fragment (Supplementary Table S1). For identification of INDEL mutation, a set of *OsGW2* gene specific primers were designed using *OsGW2* genomic sequence below and above the target sites. A 746 bp genomic region was amplified along with the knockout target site of *OsGW2* gene and PCR fragment was eluted from the agarose-gel using QIAquick gel and the PCR clean-up system (Qiagen, Germany). The PCR products (746 bp) were sequenced (Macrogen, Korea) directly using an internal gene specific sequence primer (Supplementary Table S1). The heterozygous and biallelic mutations were identified compared with its WT gene sequence using the pairwise sequence alignment bioinformatic tool MacVector 17.5 (MacVector, U.S.A.) and CRISPR-ID^63^**.**

### Visualization and assessment of aleurone morphology

Mature dehusked rice grains without visible defects from WT and *GW2*-KO lines were selected and transversally sectioned into 2–3 mm sizes using surgical blade. The thin grain sections were stained with iodine solution for 5 min, which stain starch bodies that allowed to distinguish the starchy endosperm from the aleurone layer. For visualization of the aleurone cell layer, dehusked mature grains transversally sectioned in the same manner were stained in 0.05% (w/v) Safranin for 3 min at room temperature. Following staining, thin grain sections were washed properly with milli-Q water and dried over Whatman filter paper before microscopic observations. The aleurone morphology was recorded using Zeiss SteREO Discovery V8 stereo microscope.

### Quantification of grain protein content and amino acid profiling using LC–MS/MS

Mature seeds from WT and *GW2*-KO lines were de-husked and grinded into fine powder form. About 100 mg powder was mixed with 500 ml extraction buffer Na-phosphate buffer pH 7.0 and grinded properly. Following, centrifugation at 14000xg the clear supernatant was collected and used for protein quantification. The grain protein content was quantified fluorometrically following Invitrogen Qubit Protein Assay Kits (ThermoScientific, USA) using Qubit 3 Fluorometer. BSA was used as an internal standard. For quantification of free amino acid, 20–30 dehusked rice seeds were ground into fine powder. 20 mg powder sample was mixed with 1 ml of 80% methanol and centrifuged at 4 °C. The supernatant extract was diluted in water (1:20). The diluted supernatant 40 μl was mixed with 360 μl of labelled amino acid internal standard, further processed and analysed in UPLC-MS/MS (QTRAP 6500+).

### Histochemical detection of protein, iron and zinc content in rice seed

For histochemical visualization of protein, 2–3 mm slices were soaked in the Bradford reagent (Sigma, USA) for 5 min. Following staining, the slices were thoroughly washed in MiliQ water for 15 min and dried over filter paper. Iron biochemical stain was performed using Perl’s Prussian blue (PPB) method as previously described^64^. Mature dehusked rice seed was transversely cut into 2–3 mm small pieces. The cut pieces were soaked into Prussian blue staining solution containing 1:1 (V/V) of 4% HCl and freshly prepared 4% Potassium hexacyanoferrate (II) trihydrate for 12 h. Before microscopic observation, the sliced were thoroughly washed in MiliQ water for 5 min and dried over a filter paper. The zinc determination in rice grains was performed following DTZ staining protocol^65^. Rice grains were excised longitudinally with help of scalpel, and merged in freshly-prepared DTZ solution (1,5-diphenyl thiocarbazone) at a concentration of 500 mg/l in methanol for 30 min. Half seeds were rinsed thoroughly in distilled deionized water and blotted dry using tissue paper before microscopic observation. Microscopy was performed using Carl Zeiss stereoscopic zoom microscope (Discovery V8) attached with cooled digital camera.

### Mineral quantification

For the quantification of mineral content (Fe, Zn, P, Ca and K) mature dehusked rice seeds were ground into fine powder and processed for element analysis as follows. In detail, exact 250 mg powder from each sample was mixed with 8 ml of 70% nitric acid in a digestion vial and allowed for microwave digestion at 180 °C for 20 min. After cooling, the acid hydrolysed samples were making up the volume up to 50 ml with MiliQ water and passthrough using a 0.2µ syringe filter. The samples were further diluted into 1:9 (for Fe, Zn, Ca and K) and 1:99 (for P) v/v with 2% nitric acid and allowed for ICP-MS analysis (Agilent 7800).

### Agronomy and phenotyping

The phenotypic and yield parameters such as grain width, thickness, length, plant height, number of tillers per plant, grains in the main panicle, seed weight, 100 seed weight and yield per plant were recorded at the maturity stage of the plant under containment field net house under natural conditions of ICGEB, New Delhi, India (latitude 28° 31' N, longitude 77° 10' E) during the months of June to October (temperature max 33–40 °C and min 25–28 °C). A total of 15 independent plants from each group were used for analysis. Rice seeds from WT and *GW2*-KO mutants were surface sterilized and germinated and grown hydroponically (Yoshida solution; Supplementary Table S2) in a 12-celled seedling tray filled with sterile vermiculite in the greenhouse under 14 h light (28 °C) and 10 h dark (24 °C) cycle. Seedling related data (plant biomass, root length, leaf length) were recorded on three-week (30 days) old plants.

### Statistical analysis

The quantitative experiments were performed in duplicate or triplicate with biological replications (n). All the morphological and agronomic data presented in Table 1 were recorded in the T3 generation from the T-DNA free *GW2*-KO homozygous mutant rice lines. Each data means from 15 independent plants from WT and *GW2*-KO lines. Pooled data were statistically analyzed for ANOVA (Analysis of Variance), followed by the least significant difference (LSD) test with mean ± standard deviation (SD) using Microsoft excel 2016 and GraphPad Prism 8. All phenotypic parameters were measured from the plants, grown in paddies with a distance of 20 × 20 cm under normal cultivation conditions.

### Policy statement

The collection of plant materials, the experimental research procedures, greenhouse and field studies on plants were performed in accordance with appropriate institutional, national, and international guidelines and legislation. Cultivate the plant materials and residues were discarded according to the biosafety guidelines.

## Supplementary Information

Supplementary Information 1.

Supplementary Information 2.

Supplementary Information 3.

Supplementary Information 4.
