# Supplementary material for: CRISPR-Cas9 mediated mutation in GRAIN WIDTH and WEIGHT2 (GW2) locus improves aleurone layer and grain nutritional quality in rice
Source: Sci Rep. 2021 Nov 9;11:21941. doi: 10.1038/s41598-021-00828-z (PMC8578329; doi:10.1038/s41598-021-00828-z)
Supplement: Supplementary file 1 — Supplementary Information 1. [file 41598_2021_828_MOESM1_ESM.pdf]

Supplementary material

MDYKDHDGDYKDHDIDYKDDDDKMAPKKKRKVGIHGVPAADKKYSIGLDIGTNSVGWAVITDEYKVPSKKFKVL
GNTDRHSIKKNLIGALLFDSGETAEATRLKRTARRRYTRRKNRICYLQEIFSNEMAKVDDSFFHRLEESFLVEE
DKKHERHPIFGNIVDEVAYHEKYPTIYHLRKKLVDSTDKADLRLIYLALAHMIKFRGHFLIEGDLNPDNSDVDK
LFIQLVQTYNQLFEENPINASGVDAKAILSARLSKSRRLENLIAQLPGEKKNGLFGNLIALSLGLTPNFKSNFD
LAEDAKLQLSKDTYDDDLDNLLAQIGDQYADLFLAAKNLSDAILLSDILRVNTEITKAPLSASMIKRYDEHHQD
LTLLKALVRQQLPEKYKEIFFDQSKNGYAGYIDGGASQEEFYKFIKPILEKMDGTEELLVKLNREDLLRKQRTF
DNGSIPHQIHLGELHAILRRQEDFYPFLKDNREKIEKILTFRIPYYVGPLARGNSRFAWMTRKSEETITPWNFE
EVVDKGASAQSFIERMTNFDKNLPNEKVLPKHSLLYEYFTVYNELTKVKYVTEGMRKPAFLSGEQKKAIVDLLF
KTNRKVTVKQLKEDYFKKIECFDSVEISGVEDRFNASLGTYHDLLKIIKDKDFLDNEENEDILEDIVLTLTLFE
DREMIEERLKTYAHLFDDKVMKQLKRRRYTGWGRLSRKLINGIRDKQSGKTILDFLKSDGFANRNFMQLIHDDS
LTFKEDIQKAQVSGQGDSLHEHIANLAGSPAIKKGILQTVKVVDELVKVMGRHKPENIVIEMARENQTTQKGQK
NSRERMKRIEEGIKELGSQILKEHPVENTQLQNEKLYLYYLQNGRDMYVDQELDINRLSDYDVDHIVPQSFLAD
DSIDNKVLTRSDKNRGKSDNVPSEEVVKKMKNYWRQLLNAKLITQRKFDNLTKAERGGLSELDKAGFIKRQLVE
TRQITKHVAQILDSRMNTKYDENDKLIREVKVITLKSKLVSDFRKDFQFYKVREINNYHHAHDAYLNAVVGTAL
IKKYPALESEFVYGDYKVYDVRKMIAKSEQEIGKATAKYFFYSNIMNFFKTEITLANGEIRKAPLIETNGETGE
IVWDKGRDFATVRKVLSMPQVNIVKKTEVQTGGFSKESILPKRNSDKLIARKKDWDPKKYGGFDSPTVAYSVLV
VAKVEKGKSKKLKSVKELLGITIMERSSFEKNPIDFLEAKGYKEVKKDLIIKLPKYSLFELENGRKRMLASAGE
LQKGNELALPSKYVNFLYLASHYEKLKGSPEDNEQKQLFVEQHKHYLDEIIEQISEFSKRVILADANLDKVLSA
YNKHRDKPIREQAENIIHLFTLTNLGAPAAFKYFDTTIDRKRYTSTKEVLDATLIHQSITGLYETRIDLSQLGG
DKRPAATKKAGQAKKKK*

3XFLAG sequence= DYKDHDGDYKDHDIDYKDDDDK
SV40NLS sequence=PKKKRKV
Nucleoplasmin signal sequence=KRPAATKKAGQAKKKK

Figure S1: Protein sequence of eSpCas9. The N-terminal region of eSpCas9 was fused with 3XFLAG sequence followed by SV40NLS sequence. Similarly, the C-terminal end of eSpCas9 protein was fused with Nucleoplasmin signal sequence. Three amino acid K, K and R were substituted with A to minimize the OFF-target editing and increases high level of on-target cleavage specificity.

BamH1
GGATCCATGGACTACAAGGACCACGACGGCGATTACAAGGATCATGACATCGACTATAAGGACGACGATGACAAGATGGCC
CCGAAGAAGAAGCGCAAGGTTGGCATTCATGGCGTGCCAGCCGCCGACAAGAAGTACTCTATCGGCCTCGACATCGGCACC
AACTCTGTTGGCTGGGCCGTGATCACCGACGAGTACAAGGTGCCGTCCAAGAAGTTCAAGGTCCTCGGCAACACCGACCGC
CACTCCATCAAGAAGAATCTCATCGGCGCCCTGCTGTTCGACTCTGGCGAAACAGCTGAGGCCACCAGGCTCAAGAGAACA
GCCAGACGCAGATACACCCGCCGCAAGAACAGGATCTGCTACCTCCAAGAGATCTTCTCCAACGAGATGGCCAAGGTGGAC
GACAGCTTCTTCCACCGCCTCGAGGAATCCTTCCTGGTGGAAGAGGACAAGAAGCACGAGAGGCACCCGATCTTCGGCAAC
ATCGTGGATGAGGTGGCCTACCACGAGAAGTACCCGACCATCTACCACCTCCGCAAGAAGCTCGTCGACTCCACCGATAAG
GCCGACCTCCGCCTCATCTATCTCGCCCTCGCTCACATGATCAAGTTCCGCGGCCACTTCCTCATCGAGGGCGATCTCAAC
CCGGACAACTCCGACGTGGACAAGCTGTTCATCCAGCTCGTGCAGACCTACAACCAGCTGTTCGAGGAAAACCCGATCAAC
GCCTCTGGCGTTGACGCCAAGGCTATTCTCTCTGCCAGGCTCTCTAAGTCCCGCAGGCTCGAGAACCTCATTGCTCAACTC
CCGGGCGAGAAGAAGAACGGCCTCTTCGGAAACCTGATCGCGCTCTCTCTCGGCCTCACGCCGAACTTCAAGTCCAACTTC
GACCTCGCCGAGGACGCCAAGCTCCAGCTTTCCAAGGACACCTACGACGACGACCTCGACAATCTCCTCGCGCAGATCGGC
GATCAGTACGCCGACTTGTTCCTCGCCGCCAAGAATCTCTCCGACGCCATTCTCCTCTCCGACATCCTCCGCGTGAACACC
GAGATCACAAAGGCCCCACTCTCCGCCTCGATGATCAAGAGGTACGACGAGCACCACCAGGACCTCACACTCCTCAAGGCC
CTCGTGAGACAGCAGCTCCCAGAGAAGTACAAAGAGATTTTCTTCGACCAGTCCAAGAACGGCTACGCCGGCTACATTGAT
GGCGGCGCTTCCCAAGAAGAGTTCTACAAGTTCATCAAGCCGATCCTCGAGAAGATGGATGGCACCGAGGAACTCCTCGTG
AAGCTCAACAGAGAGGACCTCCTCCGGAAGCAGCGCACCTTCGATAATGGCAGCATCCCGCACCAGATCCACCTCGGCGAA
CTCCATGCTATCCTCCGCAGGCAAGAGGACTTCTACCCGTTCCTCAAGGACAACCGCGAGAAGATTGAGAAGATCCTCACC
TTCCGCATTCCGTACTACGTGGGACCACTCGCCAGGGGCAATTCTAGGTTCGCTTGGATGACCCGCAAGTCCGAAGAGACA
ATCACCCCGTGGAACTTCGAAGAGGTGGTGGATAAGGGCGCCAGCGCGCAGTCTTTCATCGAGCGCATGACGAACTTCGAC
AAGAACCTGCCGAACGAGAAGGTGCTCCCGAAGCACTCACTCCTCTACGAGTACTTCACCGTGTACAACGAGCTGACGAAG
GTCAAGTACGTGACCGAGGGAATGCGCAAGCCAGCCTTCCTTAGCGGCGAGCAGAAAAAGGCCATCGTGGACCTGCTTTTC
AAGACCAACCGCAAGGTGACCGTGAAGCAGCTCAAAGAGGACTACTTCAAGAAAATCGAGTGCTTTGACTCCGTCGAGATC
TCCGGCGTCGAGGACAGATTCAATGCCAGCCTCGGGACGTACCACGACCTCCTCAAGATTATCAAGGATAAGGACTTCCTG
GACAACGAAGAGAACGAGGACATACTCGAGGACATCGTGCTCACGCTCACCCTCTTCGAGGACCGCGAGATGATCGAGGAA
CGCCTCAAGACATACGCCCACCTGTTCGACGACAAGGTGATGAAGCAACTCAAGCGCCGCAGGTACACAGGCTGGGGACGC
TTGTCTCGCAAGCTCATCAACGGCATCCGCGACAAGCAGTCCGGCAAGACAATCCTCGACTTCCTCAAGTCCGACGGCTTC
GCGAACCGCAACTTCATGCAGCTGATCCACGACGACTCCCTGACCTTTAAAGAGGACATCCAAAAGGCCCAGGTGTCCGGC
CAAGGCGATTCCCTCCATGAGCATATCGCCAATCTCGCTGGCTCCCCGGCCATTAAGAAGGGCATTCTCCAGACCGTCAAG
GTCGTGGACGAGCTTGTGAAGGTGATGGGCCGCCACAAGCCAGAGAACATCGTGATCGAGATGGCGCGCGAGAACCAGACC
ACACAGAAGGGCCAAAAGAACTCCCGCGAGAGGATGAAGCGCATCGAGGAAGGCATCAAAGAGCTGGGCTCCCAGATCCTC
AAAGAGCACCCAGTCGAGAACACCCAGCTCCAGAATGAGAAGCTCTACCTCTACTACCTCCAGAACGGCCGCGACATGTAC
GTGGACCAAGAGCTGGACATCAACCGCCTCAGCGACTACGATGTCGACCATATCGTGCCGCAGTCCTTCCTCGCGGACGAC
TCCATTGACAACAAGGTGCTCACCCGCTCCGATAAGAACCGCGGCAAGAGCGATAACGTGCCGAGCGAAGAGGTCGTGAAG
AAGATGAAGAACTACTGGCGGCAGCTCCTCAACGCGAAGCTGATCACCCAGCGCAAGTTCGACAACCTCACTAAGGCTGAG
AGAGGCGGCCTGTCCGAGCTTGATAAGGCGGGCTTCATCAAGAGGCAGCTCGTGGAAACCCGCCAGATCACTAAGCACGTG
GCGCAGATCCTCGACAGCCGCATGAACACCAAGTACGATGAGAACGACAAGCTGATCCGCGAGGTGAAGGTCATCACCCTC
AAGAGCAAGCTGGTGTCCGACTTCCGCAAGGATTTCCAATTCTACAAGGTCCGCGAGATCAACAACTACCACCACGCGCAC
GACGCCTACCTCAATGCCGTTGTTGGCACCGCGCTGATCAAGAAGTATCCGGCTCTCGAGTCCGAGTTCGTGTATGGCGAC
TACAAAGTGTACGACGTGCGCAAGATGATCGCCAAGTCCGAGCAAGAGATCGGCAAGGCGACCGCCAAGTATTTCTTCTAC
TCCAACATCATGAATTTCTTCAAGACCGAGATTACCCTCGCCAACGGCGAGATTAGGAAGGCCCCTCTCATCGAGACAAAC
GGCGAAACCGGCGAGATCGTGTGGGACAAAGGCAGGGATTTCGCCACGGTCCGCAAGGTCTTGTCCATGCCGCAGGTCAAC
ATCGTCAAAAAGACCGAGGTGCAGACCGGCGGCTTCAGCAAAGAGAGCATTCTCCCGAAGAGGAACAGCGACAAGCTCATT
GCCCGGAAGAAGGACTGGGACCCAAAGAAGTATGGCGGCTTCGATTCCCCGACCGTGGCCTACTCTGTTCTCGTCGTTGCC
AAGGTCGAGAAGGGCAAGTCCAAAAAGCTCAAGAGCGTGAAAGAGCTGCTCGGCATCACGATCATGGAACGCAGCAGCTTC
GAGAAGAATCCGATCGACTTTCTCGAGGCCAAGGGCTACAAAGAGGTGAAGAAGGATCTGATCATCAAGCTGCCGAAGTAC
TCCCTGTTCGAACTCGAGAATGGCCGCAAGAGGATGCTCGCTTCTGCCGGCGAGCTTCAAAAGGGCAATGAACTCGCGCTC
CCGTCCAAGTACGTCAACTTCCTCTACCTCGCCAGCCACTACGAGAAGCTCAAGGGCTCCCCAGAGGACAACGAGCAAAAG
CAACTCTTCGTCGAGCAGCACAAGCACTACCTCGACGAGATCATTGAGCAGATCTCCGAGTTCTCCAAGCGCGTGATCCTC
GCCGATGCGAACCTCGATAAGGTGCTCTCCGCGTACAACAAGCACCGCGATAAGCCAATTCGCGAGCAGGCGGAGAACATC
ATCCATCTCTTCACCCTCACCAACCTCGGCGCTCCAGCCGCCTTCAAGTACTTCGACACCACCATCGACCGCAAGCGCTAC
ACCTCCACCAAAGAGGTTCTGGACGCGACCCTGATCCACCAGTCTATCACCGGCCTGTACGAGACACGCATCGACCTCTCA
CAACTCGGCGGCGATAAGAGGCCAGCCGCAACAAAGAAAGCCGGCCAGGCCAAGAAAAAGAAGTGAGCGGCCGC
Not1

Figure S2: Rice codon optimized *eSpCas9* gene, chemically synthesized from GeneArt.

Kpn1

GGTACCAAGGAATCTTTAAACATACGAACAGATCACTTAAAGTTCTTCTGAAGCAACTTAAAGTTATCAGGCAT
GCATGGATCTTGGAGGAATCAGATGTGCAGTCAGGGACCATAGCACAAGACAGGCGTCTTCTACTGGTGCTACC
AGCAAATGCTGGAAGCCGGGAACACTGGGTACGTCGGAAACCACGTGATGTGAAGAAGTAAGATAAACTGTAGG
AGAAAAGCATTTCGTAGTGGGCCATGAAGCCTTTCAGGACATGTATTGCAGTATGGGCCGGCCCATTACGCAAT
TGGACGACAACAAAGACTAGTATTAGTACCACCTCGGCTATCCACATAGATCAAAGCTGATTTAAAAGAGTTGT
GCAGATGATCCGTGGCAGGGGAGACCCTCGAGCATGTCATTAACTTATCTTAATGTGGACAAGAAACTGATGCC
TGCTTACATTGCTATTATTTCAAGCGGGTATTGATCCTTTGACATGTGATTGATCATTTTTTTTTTCTCTGGTTA
TTAGGGCACAACAGTGGTGGACAACTTGCTGAACAGTGAGGATGTTCACTACATGCTTGAGGCCCTGAAAGCCC
TCGGGCTCTCTGTGGAAGCAGATAAAGTTGCAAAAAGAGCTGTAGTCGTTGGCTGTGGTGGCAAGTTTCCTGTT
GAGAAGGATGCGAAAGAGGAAGTGCAACTCTTCTTGGGGAACGCTGGAATTGCAATGCGAAGCTTGACAGCAGC
CGTGACTGCTGCTGGTGGAAATGCAACGTATGTTTTTTTTTTTTAATGTTTATGAAAATATGTATGGAATTCATG
GGGTATGTTTTATGACCTTTTTCTTTACCATCAGTTATGTGCTTGATGGAGTGCCACGAATGAGGGAGCGACCG
ATTGGTGACTTGGTTGTCGGGTTGAAACAACTTGGTGCGGATGTCGACTGTTTCCTTGGCACTGAATGCCCACC
TGTTCGTGTCAAGGGAATTGGAGGACTTCCTGGTGGCAAGGTTAGTTACTCCTAAACTGCATCCTTTGTACTTC
TGTATGCACCTCAATTCTTTGTCAACCTTCTGCATTTATAAGGAACATTCTATGATGCAATTCGACCTTACATC
TAGAGGTCTCG**GTTTTAGAGCTAGAAATAGCAAGTTAAAATAAGGCTAGTCCGTTATCAACTTGAAAAAGTGGC**
ACCGAGTCGGTGCTTTTTTTGTTTT**GAGCTC**

Sac1

Yellow= Rice U3 promoter

Red = *Bsa*1 site

Gray = 731bp DNA block

Underline Bold black= gRNA

Green= polIII terminator

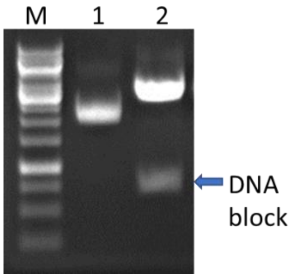

Figure S3: Expression cassette of U3-sgRNA, consisting rice U3 promoter (379bp length; yellow highlighted), sgRNA sequence (76bp length; Underline Bold black) and a DNA block (718bp length; gray color) flanked between two *Bsa*I restriction sites (highlighted red). The green highlighted sequence represents the polIII terminator sequence. The BsaI restriction digestion of U3-sgRNA expression cassette showing releasing of 731bp DNA block. Lane 1 and 2 are undigested and BsaI digested U3-sgRNA expression cassettes respectively.

Kpn1

```
GGTACCTGCAGTGCAGCGTGACCCGGTCGTGCCCCTCTCTAGAGATAATGAGCATTGCATGTCTAAGTTATAAAAAATTAC
CACATATTTTTTTTGTCACACTTGTTTGAAGTGCAGTTTATCTATCTTTATACATATATTTAAACTTTACTCTACGAATAA
TATAATCTATAGTACTACAATAATATCAGTGTTTTAGAGAATCATATAAATGAACAGTTAGACATGGTCTAAAGGACAATT
GAGTATTTTGACAACAGGACTCTACAGTTTTATCTTTTTAGTGTGCATGTGTTCTCCTTTTTTTTTGCAAATAGCTTCACC
TATATAATACTTCATCCATTTTATTAGTACATCCATTTAGGGTTTAGGGTTAATGGTTTTTATAGACTAATTTTTTTAGTA
CATCTATTTTATTCTATTTTAGCCTCTAAATTAAGAAAACTAAAACTCTATTTTAGTTTTTTTATTTAATAATTTAGATAT
AAAATAGAATAAAATAAAGTGACTAAAAATTAAACAAATACCCTTTAAGAAATTAAAAAAACTAAGGAAACATTTTTCTTG
TTTCGAGTAGATAATGCCAGCCTGTTAAACGCCGTCGACGAGTCTAACGGACACCAACCAGCGAACCAGCAGCGTCGCGTC
GGGCCAAGCGAAGCAGACGGCACGGCATCTCTGTCGCTGCCTCTGGACCCCTCTCGAGAGTTCCGCTCCACCGTTGGACTT
GCTCCGCTGTCGGCATCCAGAAATTGCGTGGCGGAGCGGCAGACGTGAGCCGGCACGGCAGGCGGCCTCCTCCTCCTCTCA
CGGCACCGGCAGCTACGGGGGATTCCTTTCCCACCGCTCCTTCGCTTTCCCTTCCTCGCCCGCCGTAATAAATAGACACCC
CCTCCACACCCTCTTTCCCCAACCTCGTGTTGTTCGGAGCGCACACACACACAACCAGATCTCCCCCAAATCCACCCGTCG
GCACCTCCGCTTCAAGGTACGCCGCTCGTCCTCCCCCCCCCCCCTCTCTACCTTCTCTAGATCGGCGTTCCGGTCCATGGT
TAGGGCCCGGTAGTTCTACTTCTGTTCATGTTTGTGTTAGATCCGTGTTTGTGTTAGATCCGTGCTGCTAGCGTTCGTACA
CGGATGCGACCTGTACGTCAGACACGTTCTGATTGCTAACTTGCCAGTGTTTCTCTTTGGGGAATCCTGGGATGGCTCTAG
CCGTTCCGCAGACGGGATCGATTTCATGATTTTTTTTGTTTCGTTGCATAGGGTTTGGTTTGCCCTTTTCCTTTATTTCAA
TATATGCCGTGCACTTGTTTGTCGGGTCATCTTTTCATGCTTTTTTTTGTCTTGGTTGTGATGATGTGGTCTGGTTGGGCG
GTCGTTCTAGATCGGAGTAGAATTAATTCTGTTTCAAACTACCTGGTGGATTTATTAATTTTGGATCTGTATGTGTGTGCC
ATACATATTCATAGTTACGAATTGAAGATGATGGATGGAAATATCGATCTAGGATAGGTATACATGTTGATGCGGGTTTTA
CTGATGCATATACAGAGATGCTTTTTGTTCGCTTGGTTGTGATGATGTGGTGTGGTTGGGCGGTCGTTCATTCGTTCTAGA
TCGGAGTAGAATACTGTTTCAAACTACCTGGTGTATTTATTAATTTTGGAACTGTATGTGTGTGTCATACATCTTCATAGT
TACGAGTTTAAGATGGATGGAAATATCGATCTAGGATAGGTATACATGTTGATGTGGGTTTTACTGATGCATATACATGAT
GGCATATGCAGCATCTATTCATATGCTCTAACCTTGAGTACCTATCTATTATAATAAACAAGTATGTTTTATAATTATTTT
GATCTTGATATACTTGGATGATGGCATATGCAGCAGCTATATGTGGATTTTTTTAGCCCTGCCTTCATACGCTATTTATTT
GCTTGGTACTGTTTCTTTTGTCGATGCTCACCCTGTTGTTTGGTGTTACTTCTGCAGGATCC
```

BamH1

Figure S4: Nucleotide sequence (1994bp) of Zea mays polyubiquitin1 promoter (ZmUbiP) amplified and sequenced from the pANIC6B vector. Highlighted region represents ZmUBI1 Intron 1.

Not1

```
GCGGCCGCCGATCGTTCAAACATTTGGCAATAAAGTTTCTTAAGATTGAATCCTGTTGCCGGTCTTGCGATGATTATCATA
TAATTTCTGTTGAATTACGTTAAGCATGTAATAATTAACATGTAATGCATGACGTTATTTATGAGATGGGTTTTTATGATT
AGAGTCCCGCAATTATACATTTAATACGCGATAGAAAACAAAATATAGCGCGCAAACTAGGATAAATTATCGCGCGCGGTG
TCATCTATGTTACTAGATCGGGAATTGAGCTC
```

Sac1

Figure S5: Nucleotide sequence (261bp) of nopaline synthase gene terminator (NosT) region amplified and sequenced pANIC6B vector.

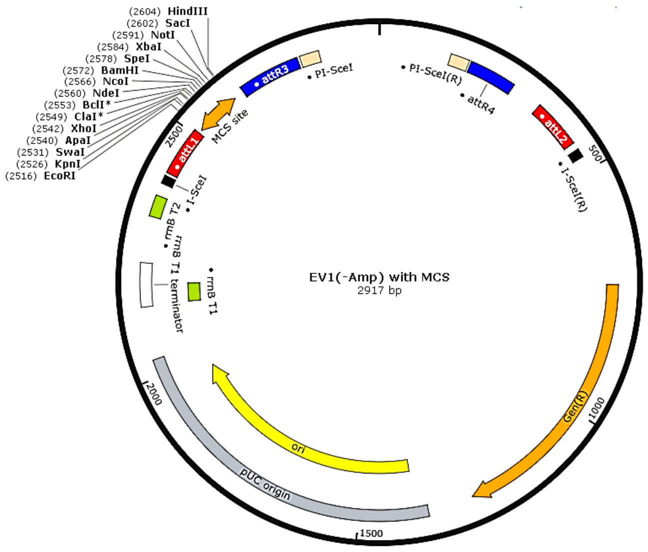

Figure S6: Vector map of pENTR(EV1; pL12R34-Amp) showing the multiple cloning sites (*EcoR*I, *Kpn*I, *Swa*I, *Apa*I, *Xho*I, *Cla*I, *Bcl*I, *Nde*I, *Nco*I, *BamH*I, *Spe*I, *Xba*I, *Not*I, *Sac*I, *Hind*III) used for the making expression cassette for cloning of promoter, gene and terminator. This image was created by SnapGene 5.1.5 software (from Insightful Science; available at https://www.snapgene.com).

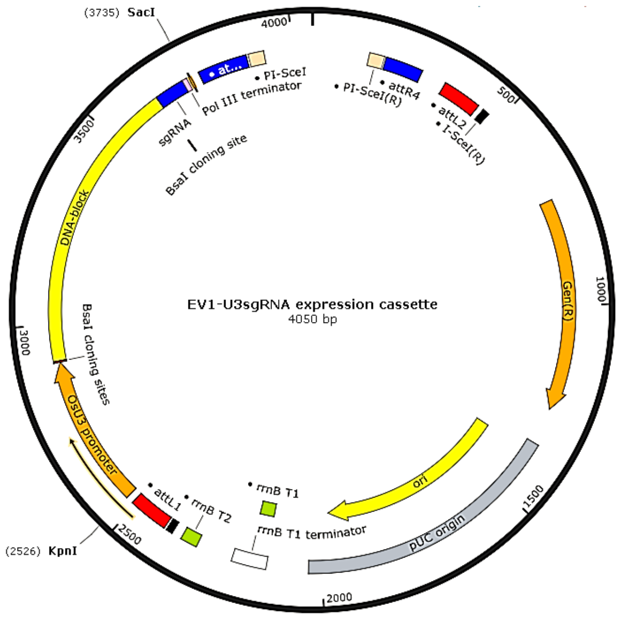

Figure S7: Vector diagram of EV1-U3sgRNA expression cassette showing rice U3 promoter, two *Bsa*I sites flanked between a DNA block, sgRNA and PolIII terminator. The chemical synthesized U3sgRNA expression cassette was cloned in the *Kpn*I and *Sac*I sites in the EV1 vector. This image was created by SnapGene 5.1.5 software (from Insightful Science; available at https://www.snapgene.com).

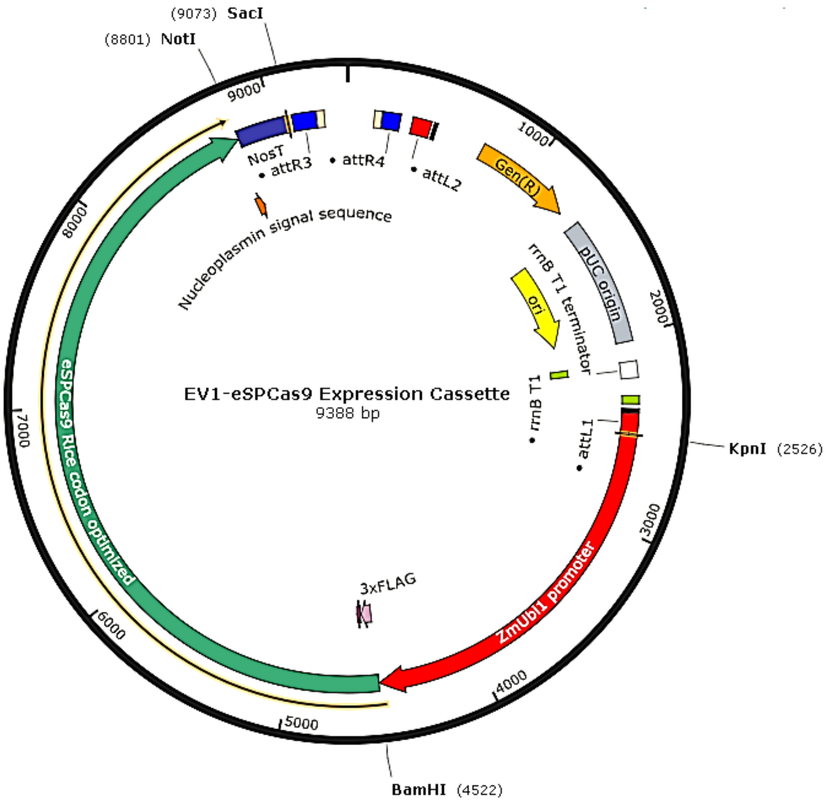

Figure S8: Vector diagram showing expression cassette of *eSpCas9* gene in EV1. The *Zea mays* ubiqutin1 promoter (ZmUbi1P; 1994bp, cloned *Kpn*I and *BamH*I), *eSpCas9* gene (*eSpCas9*; 4272bp, cloned *BamH*I and *Not*I) and nopaline synthase terminator (NosT; 261bp, cloned *Not*I and *Sac*I) was cloned into the sequentially in the polylinker sits of EV1. This image was created by SnapGene 5.1.5 software (from Insightful Science; available at https://www.snapgene.com).

GW2-KO1 (C-Insertion)

```
GW2-wt   :MGNRIGGRRKAGVEERYTRPQGLYEHRDIDQKKLRKLILEAKLAPCYMGADDAAAAADLEECPICFLYYPSLNRSKCCSKGICTECFLQM 90
GW2-KO1  :MGNRIGGRRKAGVEERYTRPQGLYEHRDIDQKKLRKLILEAKLAPCYMGADDAAAAADLEECPICFLYYPSLNRSKCCSKGICTECFLQM 90
          ******************************************************************************************

GW2-wt   :KPTHTAQPTQCPFCKTPSYAVEYRGVKTKEERSIEQFEEQKVIEAQMRMRQQALQDEEDKMKRKQNRCSSSRTITPTKEVEYRDICSTSF 180
GW2-KO1  :KPTHTAQPTQCPFCKTPSYAVEYSWCKDKGGKEHRTI*..................................................... 127
          ***********************

GW2-wt   :SVPSYRCAEQETECCSSEPSCSAQTSMRPFHSRHNRDDNIDMNIEDMMVMEAIWRSIQGSIGNPVCGNFMPVTEPSPRERQPFVPAASLE 270
GW2-KO1  :..........................................................................................

GW2-wt   :IPHGGGFSCAVAAMAEHQPPSMDFSYMAGSSAFPVFDMFRRPCNIAGGSMCNLESSPESWSGIAPSCSREVVREEGECSADHWSEGAEAG 360
GW2-KO1  :..........................................................................................

GW2-wt   : TSYAGSDIVADAGTMPQLPFAENFAMAPSHFRPESIEEQMMFSMALSLADGHGRTHSQGLAWL*
GW2-KO1  :.................................................................
```

GW2-KO2 (A-Deletion)

```
GW2-wt   :MGNRIGGRRKAGVEERYTRPQGLYEHRDIDQKKLRKLILEAKLAPCYMGADDAAAAADLEECPICFLYYPSLNRSKCCSKGICTECFLQM 90
GW2-KO2  :MGNRIGGRRKAGVEERYTRPQGLYEHRDIDQKKLRKLILEAKLAPCYMGADDAAAAADLEECPICFLYYPSLNRSKCCSKGICTECFLQM 90
          ******************************************************************************************

GW2-wt   :KPTHTAQPTQCPFCKTPSYAVEYRGVKTKEERSIEQFEEQKVIEAQMRMRQQALQDEEDKMKRKQNRCSSSRTITPTKEVEYRDICSTSF 180
GW2-KO2  :KPTHTAQPTQCPFCKTPSYAVEFVV*...............................................................   127
          **********************

GW2-wt   :SVPSYRCAEQETECCSSEPSCSAQTSMRPFHSRHNRDDNIDMNIEDMMVMEAIWRSIQGSIGNPVCGNFMPVTEPSPRERQPFVPAASLE 270
GW2-KO2  :..........................................................................................

GW2-wt   :IPHGGGFSCAVAAMAEHQPPSMDFSYMAGSSAFPVFDMFRRPCNIAGGSMCNLESSPESWSGIAPSCSREVVREEGECSADHWSEGAEAG 360
GW2-KO2  :..........................................................................................

GW2-wt   : TSYAGSDIVADAGTMPQLPFAENFAMAPSHFRPESIEEQMMFSMALSLADGHGRTHSQGLAWL*
GW2-KO2  :.................................................................
```

Figure S9: Effect of indel point mutation in the *OsGW2* locus resulted formation of truncated protein. Rice *GW2* gene encodes for a 423 amino acid polypeptide. *GW2*-KO1 having C insertion leads to introduction of premature stop codon at 127 amino acid position. Similarly, *GW2*-KO2 having A-deletion mutation terminated with a stop codon at 115 amino acid position.
