# Supplementary material for: CRISPR-Cas9 mediated mutation in GRAIN WIDTH and WEIGHT2 (GW2) locus improves aleurone layer and grain nutritional quality in rice
Source: Sci Rep. 2021 Nov 9;11:21941. doi: 10.1038/s41598-021-00828-z (PMC8578329; doi:10.1038/s41598-021-00828-z)
Supplement: Supplementary file 2 — Supplementary Information 2. [file 41598_2021_828_MOESM2_ESM.pdf]

Table S1 Primer used in the present study.

	Primer name	5'-NNNNNNNN-------NNNNNNN-3'
	GW2-20nt Target-F	CAGGAGTTATGCTGTGGAGTATCG
	GW2-20nt Target-R	AAACCGATACTCCACAGCATAACT
	GW2 Screen F	CCACTCCTGTCCTGAAATGC
	GW2 Screen R	ACATCGGTATGACGGCACTG
	GW2 Seq F	ACCGATCAAAGTGTTGCTCAAA
	OsU3 Screen-F	GTGCAGTCAGGGACCATAGCA
	ZmUbiP-Screen F	GTTGGGCGGTCGTTCATTCGTTC
	Cas9-Screen R	GAAGATCGGGTGCCTCTCGT
	hpt Screen F	GCCTGAACTCACCGCGACG
	hpt Screen R	CAGCCATCGGTCCAGACG
	ZmUbiP (*Kpn*I) F	ATAGGTACCTGCAGTGCAGCGTGACCCGGT
	ZmUbiP (*BamH*I) R	AGGATCCTGCAGAAGTAACACCAAACAAC
	NosT (*Not*I)F	ATTGCGGCCGCCGATCGTTCAAACATTTGGC
	NosT (*Sac*I)R	TAAGAGCTCAATTCCCGATCTAGTAACATAGATG
