# Supplementary material for: CRISPR-Cas9 mediated mutation in GRAIN WIDTH and WEIGHT2 (GW2) locus improves aleurone layer and grain nutritional quality in rice
Source: Sci Rep. 2021 Nov 9;11:21941. doi: 10.1038/s41598-021-00828-z (PMC8578329; doi:10.1038/s41598-021-00828-z)
Supplement: Supplementary file 3 — Supplementary Information 3. [file 41598_2021_828_MOESM3_ESM.pdf]

Table S2 Yoshida stock solution composition.

Major Salt	gm/l (stock)	working	Minor Salt	gm/l	working
NH_4NO_3	91.4	1.25 ml	$MnCl_2 . 4H_2O$	1.5	1.25 ml
K_2SO_4	71.4	1.25 ml	$(NH_4)_6 Mo_7 O_{24} . 4H_2O$	0.074	
KH_2PO_4	23.1	1.25 ml	H_3BO_3	0.934	
K_2HPO_4	04.3	1.25 ml	$ZnSO_4 . 7H_2O$	0.035	
$CaCl_2 . 2H_2O$	117.4	1.25 ml	$CuSO_4 . 7H_2O$	0.031	
$MgSO_4 . 7H_2O$	324.0	1.25 ml	$FeCl_3 . 6H_2O$	7.7	
			$C_6H_8O_7 . H_2O$	11.9	
			H_2SO_4	50ml	

Yoshida working solution composition:
For making one liter Yoshida working solution add 1.25 ml of each component of major salt and 1.25ml from the minor salt mixture. Adjust the pH of the final working solution to 5.8 using KOH solution.
