# Supplementary material for: CRISPR-Cas9 mediated mutation in GRAIN WIDTH and WEIGHT2 (GW2) locus improves aleurone layer and grain nutritional quality in rice
Source: Sci Rep. 2021 Nov 9;11:21941. doi: 10.1038/s41598-021-00828-z (PMC8578329; doi:10.1038/s41598-021-00828-z)
Supplement: Supplementary file 4 — Supplementary Information 4. [file 41598_2021_828_MOESM4_ESM.pdf]

Unprocessed gels for Figure 1E

Cas9 **screen**

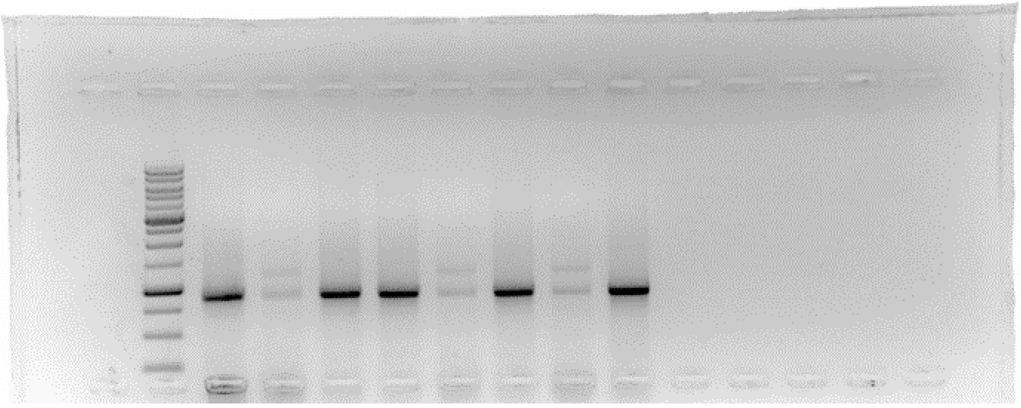

hpt **screen**

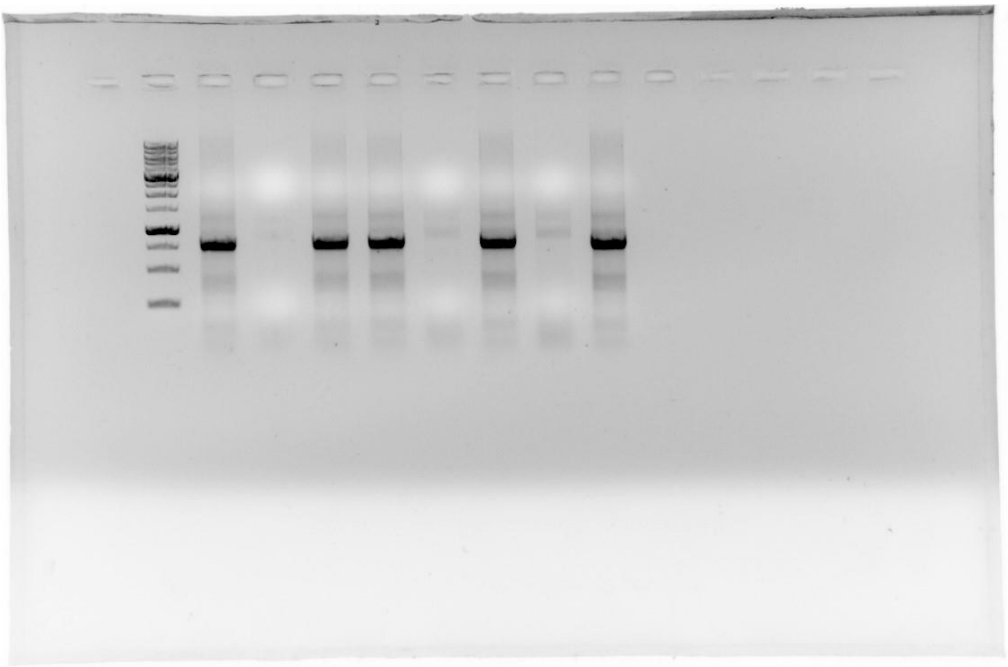

Target screen

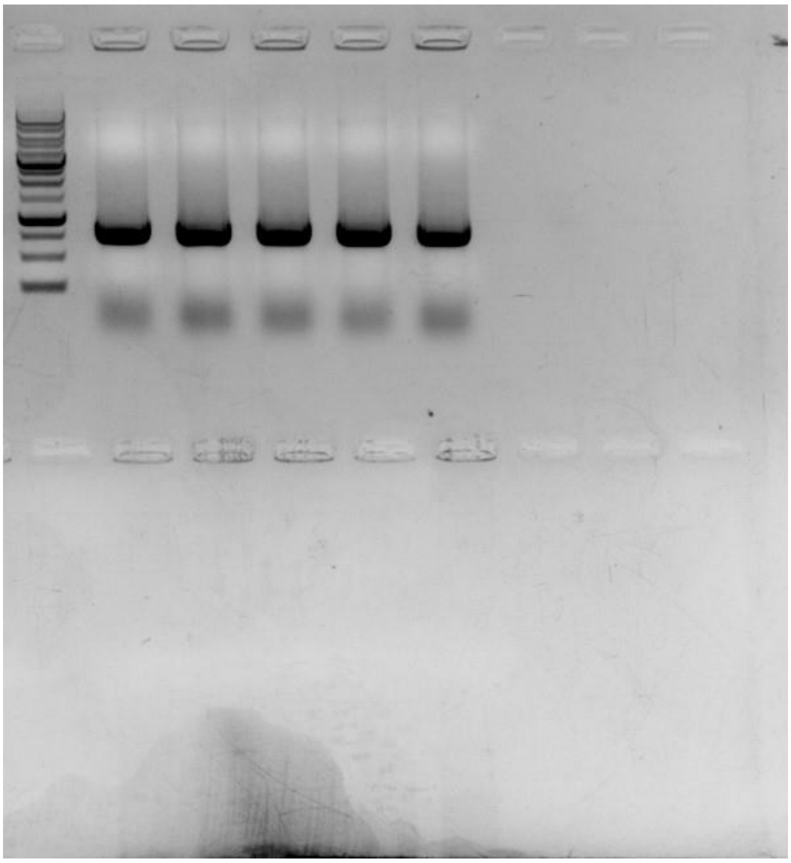

Unprocessed gels for Supplementary Figure 3

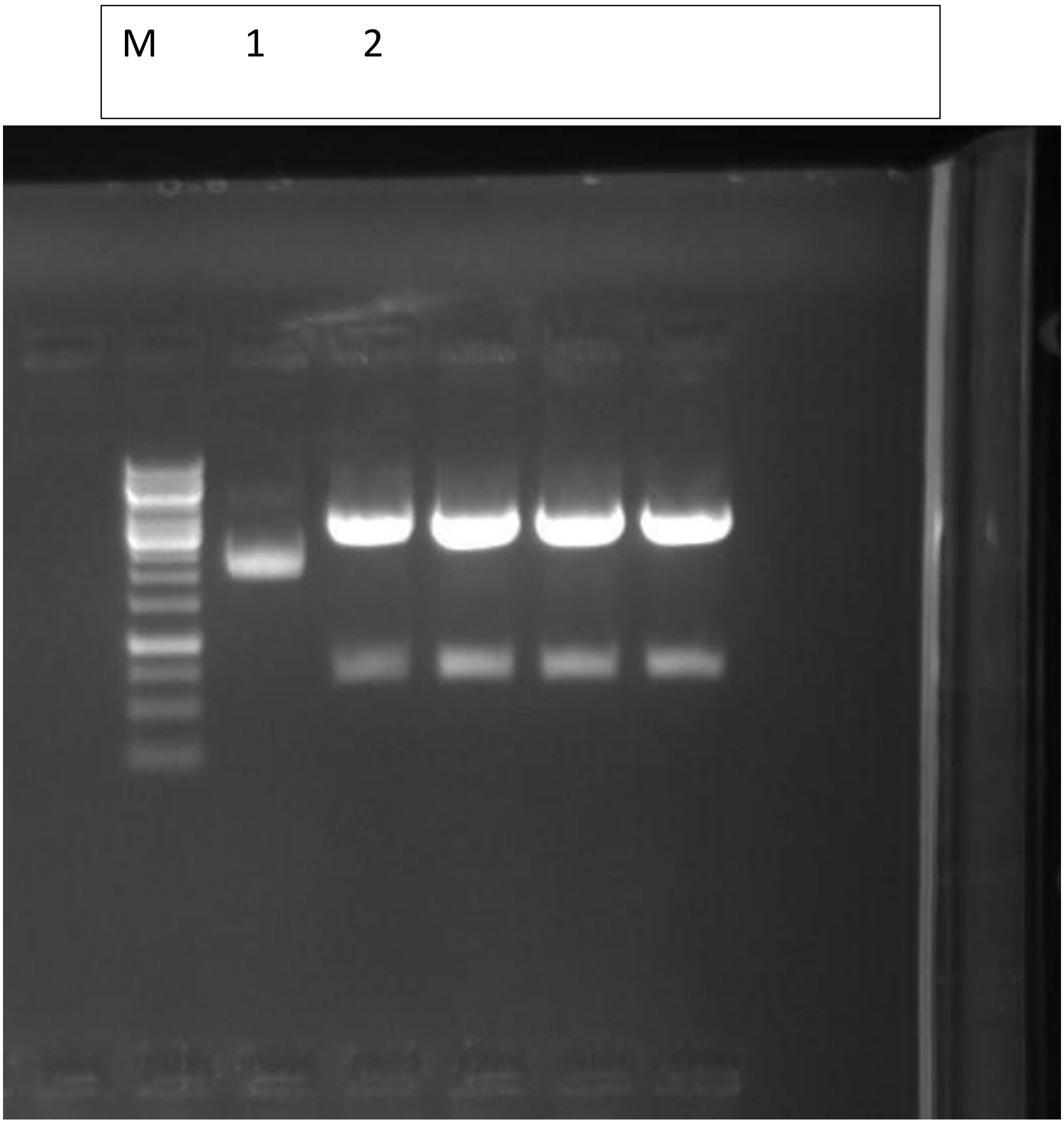

The *Bsa*I restriction digestion of U3-sgRNA expression cassette showing releasing of 731bp DNA block. Lane 1 and 2 are undigested and *Bsa*I digested U3-sgRNA expression cassettes respectively.
